# *In silico* characterization, molecular phylogeny, and expression profiling of genes encoding legume lectin-like proteins under various abiotic stresses in *Arabidopsis thaliana*

**DOI:** 10.1186/s12864-022-08708-0

**Published:** 2022-06-29

**Authors:** Subhankar Biswas, Raju Mondal, Akanksha Srivastava, Maitri Trivedi, Sunil Kumar Singh, Yogesh Mishra

**Affiliations:** 1 Department of Botany, Centre of Advanced Study in Botany, Institute of Science, Banaras Hindu University, 221005 Varanasi, Uttar Pradesh India; 2 Mulberry Tissue Culture Lab, Central Sericultural Germplasm Resources Center, Central Silk Board-Ministry of Textiles (GoI), 635109 Hosur, Tamil Nadu India; 3 Plant Cell and Molecular Biology Lab, Department of Botany, Faculty of Science, The Maharaja Sayajirao University of Baroda, 390 002 Vadodara, Gujarat India

**Keywords:** Abiotic stress, *Arabidopsis thaliana*, Legume lectin-like proteins, Receptor-like kinases, Signaling

## Abstract

**Background:**

Lectin receptor-like kinases (Lec-RLKs), a subfamily of RLKs, have been demonstrated to play an important role in signal transduction from cell wall to the plasma membrane during biotic stresses. Lec-RLKs include legume lectin-like proteins (LLPs), an important group of apoplastic proteins that are expressed in regenerating cell walls and play a role in immune-related responses. However, it is unclear whether LLPs have a function in abiotic stress mitigation and related signaling pathways. Therefore, in this study, we examined the possible role of LLPs in *Arabidopsis thaliana* (AtLLPs) under various abiotic stresses.

**Results:**

The study was initiated by analyzing the chromosomal localization, gene structure, protein motif, peptide sequence, phylogeny, evolutionary divergence, and sub-cellular localization of AtLLPs. Furthermore, the expression profiling of these *AtLLPs* was performed using publicly accessible microarray datasets under various abiotic stresses, which indicated that all *AtLLPs* were differently expressed in both root and shoot tissues in response to abiotic stresses. The *cis*-regulatory elements (CREs) analysis in 500 bp promoter sequences of *AtLLPs* suggested the presence of multiple important CREs implicated for regulating abiotic stress responses, which was further supported by expressional correlation analysis between *AtLLPs* and their CREs cognate transcription factors (TFs). qRT-PCR analysis of these AtLLPs after 2, 6, and 12 h of cold, high light, oxidative (MV), UV-B, wound, and ozone stress revealed that all AtLLPs displayed differential expression patterns in most of the tested stresses, supporting their roles in abiotic stress response and signaling again. Out of these *AtLLPs*, AT1g53070 and AT5g03350 appeared to be important players. Furthermore, the mutant line of AT5g03350 exhibited higher levels of ROS than wild type plants till 12 h of exposure to high light, MV, UV-B, and wound, whereas its overexpression line exhibited comparatively lower levels of ROS, indicating a positive role of this gene in abiotic stress response in *A*. *thaliana*.

**Conclusions:**

This study provides basic insights in the involvement of two important representative *AtLLPs*, AT1g53070 and AT5g03350, in abiotic stress response. However, further research is needed to determine the specific molecular mechanism of these *AtLLPs* in abiotic stress mitigation and related signaling pathways in *A*. *thaliana*.

**Supplementary Information:**

The online version contains supplementary material available at 10.1186/s12864-022-08708-0.

## Background

Plants are sessile and frequently exposed to various external factors, including biotic and abiotic stresses that adversely affect their growth and productivity. The perception and transmission of these stresses are regulated by sensors and downstream signaling components, including receptor-like kinases (RLKs) [1–4]. More than 600 RLK genes have been identified in the *Arabidopsis thaliana* genome, with many of them being classified into subfamilies depending on their extracellular domains [1]. RLKs extracellular domain diversity shows a wide range of activities and signal transduction modalities. To completely understand the functions of RLKs, it is important to know about their interacting ligands and their downstream targets. The adaptive responses of plants to extracellular ligands and stimuli are regulated by a functional continuity between the cell wall (CW) and plasma membrane (PM) in which RLKs with CW-bound extracellular domains presumably serve as cell wall integrity sensors [3]. The lectin-receptor-like kinases (Lec-RLKs), a subfamily of RLKs with extracellular lectin motifs that bind various carbohydrates, are believed to be possible CW-PM linkers. Lec-RLKs have an important role in both plant development and stress responses. They have been linked to seed germination, lateral root growth, pollen development, hormone signaling, defenses against pathogens, and insect pests [2, 5–11]. Moreover, there is certain evidence on their probable functions in abiotic stresses [12, 13].

The lectin motif has been reported to be involved in stress signaling, development, and defense in plants [14]. As per their lectin domain-type, Lec-RLKs are classified in three groups: GNA-related lectins (G-type Lec-RLKs), legume lectins (L-type Lec-RLKs), and Ca-dependent lectins (C-type Lec-RLKs) [15, 16].

L-type Lec-RLKs are one of the most important groups of Lec-RLKs in *A. thaliana*; moreover, they are known to play an important role in plant development and defense [17]. The important features of L-type Lec-RLKs are the presence of signal peptide (SP), L-type lectin domain, transmembrane domain, and intracellular kinase domain. In *A. thaliana*, certain L-type Lec-RLKs are referred to as legume lectin-like proteins (LLPs) because they lack the transmembrane (TM) and kinase domains [1, 18]. As members of the L-type Lec-RLK group, LLPs may function as CW-PM linkers and cell wall integrity sensors during abiotic stress mitigation/signaling. However, their biological significance in *A. thaliana* has to yet be determined.

In *A. thaliana* and certain other Brassicaceae species, LLPs have been examined at the transcriptional and proteomic level in response to many biotic stresses. The proteomic analysis of cell wall regeneration in *A. thaliana* protoplast suspension culture demonstrated that certain LLPs, including AT1g53070, AT3g16530, and AT3g15356, may play an important role in cell wall organization or regeneration [19]. Another *LLP*, AT5g03350, was reported to be among the top expressed genes in *A. thaliana* after treatment with salicylic acid [20]. Armijo et al. (2013) reported its induction in *A. thaliana* against *Pseudomonas syringae* and named as *salicylic acid induced legume lectin like protein 1 (SAI-LLP1)* [21]. Moreover, the up-regulation of this gene was reported in *A. thaliana* after infection with the cucumber mosaic virus (CMV(Y) [22]. These results show that AT5g03350 provides protection against pathogen attack. Certain hormonal regulations such as cytokinins [23] and sritinol, an artificial inducer for auxin inducible genes [24], have been investigated on this LLP. In *A. thaliana*, AT3g15356 also demonstrated increased expression after infection with *Alternaria brassicicola* [25]. In addition, expressional analysis of *Brassica* LLPs revealed that they are up-regulated in response to *Sclerotinia sclerotiorum* and *Pieris rapae* infection/attack [26, 27]. The increased expression of *LLPs* was detected in regenerated cell walls, indicating their significance in stress-induced cell wall rearrangement [19, 28, 29]. Another *LLP*, AT3g16530, demonstrated downregulation in *A. thaliana* after arsenic related stress [30], which is unlike the behavior of the aforementioned LLPs under biotic stresses.

To summarize, the abovementioned studies demonstrated that LLPs are related to various biotic stresses that are induced by pathogens and phytohormones [20, 21, 23, 25]. Since abiotic stresses may induce cell wall rearrangement and alter pH, redox, and osmotic balance, there is a possibility that LLPs are participating in abiotic stress mitigation and related signaling pathways. However, how involved LLPs are in abiotic stress mitigation/signaling is not very clear.

Therefore, in this study, we examined the possible role of LLPs in *A. thaliana* (hereafter *AtLLPs*) in response to several abiotic stresses. The gene sequences of *AtLLPs* were first retrieved, and then their chromosomal localization, gene structure, phylogeny, and sub-cellular localization was obtained. The evolutionary divergence of this gene family in the course of evolution was analyzed by comparing evolutionary rate ratio (dN/dS) of *AtLLPs* and its orthologous pairs in which dN corresponds to non-synonymous substitutions per non-synonymous site and dS corresponds to synonymous substitutions per synonymous site. Furthermore, the expression patterns of these genes were analyzed using publicly accessible microarray datasets from both root and shoot tissues under multiple abiotic stresses. The *cis*-regulatory elements (CREs) and their associated transcription factors (TFs) were identified in the promoter regions of *AtLLPs* to better understand their mechanism of transcriptional regulation and identify important regulatory TFs that may affect these gene expressions. Furthermore, the expression patterns of these *AtLLP* genes were examined using quantitative-reverse transcription (qRT-PCR) after 2, 6, and 12 h duration of cold, high light, oxidative (MV), UV-B, wound, and ozone stress. To characterize the function of AT5g03350, one of the key *AtLLPs* under tested abiotic stresses, we subsequently evaluated the abiotic stress response in terms of ROS generation in AT5g03350 mutant (T-DNA insertion SALK_036814 line), 35 S::AT5g03350 overexpression and wild type plants after 2, 6, and 12 h of cold, high light, oxidative, UV-B, wound, and ozone stress.

The results of this study will increase our understanding of the involvement of *AtLLPs* in abiotic stresses and related signaling transduction pathways; moreover, they will provide a solid base for the further functional characterization of *AtLLP* genes in *A. thaliana*.

## Methods

### Gene identification and genome-wide physical localization of *AtLLPs*

*AtLLPs* were identified on the basis of previous studies [1, 18] and the physical localization of this group of genes across the complete genome of *A. thaliana* was identified using TAIR chromosomal map tools (www.arabidopsis.org). In *A. thaliana*, seven *AtLLPs*, including AT1g07460, AT1g53060, AT1g53070, AT1g53080, AT3g16530, AT5g03350, and AT3g15356 have been identified in total [1].

### Gene structure, protein motif, peptide sequence alignment, phylogenetic relationship, and physicochemical analyses of AtLLPs

The gene sequences of *AtLLPs* were retrieved from TAIR (https://www.arabidopsis.org/) and their gene structures were analyzed using TBTools 0.665 (https://github.com/CJ-Chen/TBtools) [31]. To predict the functional motif of AtLLPs, protein sequences were downloaded from TAIR and submitted for online MEME suit analysis (https://meme-suite.org/meme/). The peptide sequence alignment of AtLLPs was performed in Jalview using the Clustal Omega program [32]. To develop the phylogenetic tree, the orthologs of AtLLPs present in *Marchantia polymorpha, Physcomitrella patens, Selaginella moellendorffii, Amborella trichopoda, Arabidopsis lyrata, Brassica oleracea, Brassica rapa, Daucus carota, Solanum lycopersicum, Solanum tuberosum, Oryza sativa ssp. japonica, Zea mays, Triticum aestivum*, and *Populus trichocarpa* were identified using PLAZA 5.0 [33]. Their peptide sequences were retrieved from NCBI and Phytozome [34]. Peptide sequences were then aligned using the ClustalW program in MEGA 11. The neighbour-joining tree was constructed using 1000 bootstrap replicates, and the physicochemical characterization of protein sequences was performed using ProtParam (https://web.expasy.org/protparam/), a peptide sequence analysis tool [35].

### Sub-cellular localization prediction of AtLLPs

Sub-cellular localization of AtLLP proteins was analyzed using SUBcellular localization database of Arabidopsis proteins (SUBA4, https://suba.plantenergy.uwa.edu.au/) [36].

### Orthologs identification and evolutionary divergence analysis of *AtLLPs*

Orthologs of six *AtLLPs* (except At1G07460.1, which appears to be an outlier among the other *AtLLPs*) were retrieved from genome database of *Arabidopsis lyrata, Brassica rapa*, *Solanum lycopersicum*, *Zea mays*, *Selaginella moellendorffii, Physcomitrium patens*, and *Chlamydomonas reinhardtii* using NCBI genome database. Confirmation of orthologous relationship was done through phylogenetic tree construction using UPGMA method in MEGAX [37]. dN (non-synonymous substitutions per non-synonymous site) and dS (synonymous substitutions per synonymous site) values of *AtLLPs* and its orthologous pairs were calculated using PAL2NAL (http://www.bork.embl.de/pal2nal/), which has codeml program of PAML package [38]. The dS values of gene pairs so obtained were used to estimate their evolutionary divergence using formula T = dS/(2 × 6.5 × 10 − 9)×10 − 6 MYA. Divergence time of *AtLLPs* and its orthologs from plant family was compared with evolutionary time scale of speciation events (http://www.timetree.org/) [39].

### *In silico* expression analysis of *AtLLPs* under various abiotic stress conditions

Publicly accessible microarray-based gene expression data were used for the *in silico* expression investigation of six *AtLLPs* under diverse abiotic stressors, with the exception of AT3g15356, which lacks expression data in the database. Nine abiotic stress conditions, including cold, osmotic, salt, drought, genotoxic, oxidative, UV-B, wounding, and heat stress, were selected to examine the expression of *AtLLPs*. Therefore, nine AtGeneExpress (Stress Series) samples were retrieved from Bio-Analytic Resource, e-Northerns w. Expression Browser (http://bar.utoronto.ca; [40], and the array was normalized with a TGT value of 100 using the Gene Chip Operating Software (GCOS). The datasets comprised an average of replicate treatments compared to the average of appropriate control and output values in the table and image were log2-transformed ratios. For differentially expressed genes (DEG) analysis, datasets comprised seven data points of nine samples that were extracted in notepad, and then the differential expression of six selected genes was depicted in a heatmap using default criteria of the TBtools software version 0.665 [31]. The experimental conditions for the extracted expression data from Bio-Analytic Resource, e-Northerns w. Expression Browser (http://bar.utoronto.ca; [40]) were as follows: *A. thaliana* wild-type (col-0) seeds were spread in magenta boxes containing the Murashige and Skoog (MS) medium. After two days of incubation at 4 °C in dark, the samples were transferred to the growth chamber (photoperiod of 16/8 h, temperature of 24 °C, relative humidity of 50%, and light intensity of 150 µE/cm^2^ s). The above-mentioned stresses were applied to 16-day-old seedlings for 0.5, 1.0, 3.0, 6.0, 12.0, and 24.0 h, with control samples at 0 h. For tissue-specific expression, roots and shoots were separated, and all treatments were performed on the same batch of seedlings.

### Promoter analysis, functional annotation of *cis*-regulatory elements (CREs), and identification of cognate TFs in *AtLLPs*

To understand the promoter structure and distribution of CREs, 500 bp non-coding sequences that were upstream of the transcription start site (TSS) of candidate genes were retrieved from TAIR and analyzed in PlantRegMap (http://plantregmap.gao-lab.org/) [41]. The stress-related CREs was identified and displayed using TBtools 0.665 [31]. Cognate TFs that bind to CREs at the promoter regions of *AtLLPs* were selected from PlantRegMap (http://plantregmap.gao-lab.org/) [41].

### Expressional correlation analysis between *AtLLPs* and their CREs cognate TFs

The normalized expression data for *AtLLPs* and their CREs cognate TFs was retrieved from e-Northerns w. Expression Browser as described in the *in silico* expression analysis of *AtLLPs* under similar nine abiotic stress conditions. For six AtLLPs, the TFs were chosen based on the availability of their expression in the dataset. The seventh *AtLLP*, AT3g15356, is likewise not included in this analysis due to a lack of expression data in the database. The normalized expression data of each *AtLLPs* was plotted against respective TFs data, and the Pearson correlation coefficient (r) value was calculated. R values of > 0.8 and < − 0.8 were considered as significant. TFs that show significant expression correlation with corresponding *AtLLPs* at least in a single stress condition was plotted as heatmap using Morpheus (https://software.broadinstitute.org/morpheus, version 1.0).

### Plant material, growth condition and abiotic stress treatment

The seeds of *A. thaliana* ecotype Columbia-0 (Col-0) were obtained from the Arabidopsis Biological Resource Centre (ABRC), The Ohio State University, USA. Seeds were sterilized for 10 min in a solution of freshly prepared 5% calcium hypochorite and 0.02% Triton-x 100. After being washed 4–5 times with sterilized water, seeds were plated on half-strength MS medium with 1% sucrose and 0.8% plant agar. The plates were then maintained at 4 °C for three days to achieve synchronized and higher seed germination. Then, plates were placed in a plant growth chamber (Conviron, Adaptis 1000) at 23 °C, bright fluorescent light of 150 µmol photons m^− 2^ s^− 1^, relative humidity of 65% and 16 h light: 8 h dark cycle.

For stress treatment, 10-day-old seedlings were subjected to six abiotic stresses, namely, cold (8 °C), high light (500 µmol photons m^− 2^ s^− 1^), oxidative (25 µM, MV), UV-B (0.99 W m^− 2^ s^− 1^), wound, and ozone (40 ppb) for a duration of 2, 6, and 12 h in triplicates, as previously described by Mondal et al. (2021) [4]. In addition to each stress treatment, three similar sets of seedlings were maintained in their normal growing condition and used as a control.

### RNA isolation and cDNA synthesis

Total RNA was isolated from stress-exposed seedlings at selected time points, including 0 (control), 2, 6, and 12 h using the RNeasy plant mini kit (Qiagen, USA) as per manufacturer’s instructions. The quantity and quality of isolated RNA were determined by spectrophotometry (Nanodrop 2000, Thermo Fisher Scientific, Waltham, MA, USA) and formaldehyde-based gel electrophoresis, respectively (Additional File 1: Fig S1). For cDNA synthesis, 1 µg of total RNA was reverse-transcribed in 20 µl using Revert Aid First Strand cDNA Synthesis Kit (Fermentas Life Sciences, USA) and oligo (*dT*) primers according to the manufacturer’s instructions.

### Expression analysis of *AtLLPs* under six selected abiotic stresses

To examine the temporal expression patterns of *AtLLPs* under six different abiotic stresses, qRT-PCR was performed at 2, 6, and 12 h of exposure to cold, high light, oxidative, UV-B, wound, and ozone stress. qRT-PCR was performed in CFX-96 Real-time PCR Detection System (Bio-Rad, USA). Reactions were conducted in a total volume of 20 µl using 50 ng of cDNA, 0.5 to 1 pmol of forward and reverse primers, and 10 µl of 2x Sso Fast Eva Green qPCR Supermix (Bio-Rad, USA). The cycling conditions were as per the manufacturer’s protocol with a primer-specific annealing temperature. The threshold cycle (C_t_) was automatically determined for each reaction using the system set with default parameters. The transcript levels were normalized to *AtUBQ5* and *AtAPT1* transcript and the fold differences of each amplified product in the samples was calculated using the 2^−ΔΔCt^ method. All primer sequences used in this study were designed using the NCBI-Primer blast online tool (https://www.ncbi.nlm.nih.gov/tools/primer-blast/) and are listed in Additional File 2: Table S1.

### Vector construction, generation of At5g03350::overexpression plants and selection of homozygous lines

Gateway cloning strategy was employed to clone the CDS of AT5g03350. Primers were design with Vector NTI Software (ThermoFischer scientific) with flanking attB1 and attB2 sequences (Additional File 2: Table S1). *attB*-flanked PCR products were amplified in 25 µl PCR reactions that contained 5 µl of 5X Phusion HF Buffer (Thermo scientific), 0.5 µl of 10 mM dNTP mix (Thermo-R0191), 0.5 µl of 10 mM forward and reverse primers, 100 ng (1 µl) cDNA, 0.25 µl of Phusion™ DNA Polymerase (2 U µl^− 1^), and 17.25 µl nuclease free water (Sigma). Amplified PCR product of 885 bp was purified using QIAquick gel extraction kit (QIAGEN, cat. no. 28,704) as per the manufacturer instruction (Additional File 3: Fig. S2 A, B).

To generate entry clone, BP reaction was performed, in which 2 µl (75 ng) attB-flanked PCR product, 1 µl (150 ng) pDONR207 (Invitrogen, USA), 5 µl of TE buffer, and 1 µl Gateway 2 µl of BP Clonase™ II enzyme (Invitrogen, USA) were mixed and incubation at 25 °C for 1 h. The reaction was terminated with the addition of 1 µl Protease K and incubation at 37 °C for 15 min. Subsequently 1 µl of BP reaction was transformed in DH5-alpha competent cells. Transformed colonies were selected using gentamycin (10 µg ml^− 1^) on LB-plates. The transformed colonies were PCR verified for AT5g03350:CDS using same primers used for CDS amplification (Additional File 3: Fig. S2 C, D). The entry clone AT5g03350 CDS- pDONR207 plasmid was isolated using the Qiagen Plasmid mini kit (catalogue no. 12,123) according to the manufacturer’s instructions and further confirmed by DNA sequencing using the ATTL1 and ATLL2 primers (Additional File 2: Table S1; Additional File 4 and 5). Subsequently expression clone was generated by LR reaction which include 1 µl (100 ng µl^− 1^) entry clone (AT5g03350 CDS-pDONR207), 1 µl (150 ng µl^− 1^) pMDC32 destination vector [42] 4 µl TE buffer and 2 µl LR Clonase II Enzyme mix (Invitrogen). The reaction mixture was incubated as described for BP reaction. The LR reaction was transformed in DH5-alpha competent cells and positive colonies were selected on 50 µg ml^− 1^ kanamycin. The selected colonies were cross-validated using PCR as described for BP reaction (Additional File 3: Fig S2 C, D). The 35 S::AT5g03350 CDS-pMDC32 clone was isolated using Qiagen Plasmid mini kit (cat. No. 12,123) and used for transformation of *Agrobacterium tumefaciens* GV3101 cells.

Electroporation strategy was used to transform the *Agrobacterium* cells. 100 µL electrocompetent *Agrobacterium* GV3101 (helper plasmid pMP90GR) cells and 50–100 ng of 35 S::AT5g03350 CDS-pMDC32 clone were mixed and placed in electroporation cuvette (Gene Pulser Cuvettes, Bio-Rad) on ice for 30 min. Gene pulser (Bio-Rad) was set to 2.5 kV, 200 Ω, and 25 µFD. Cuvette containing *Agrobacterium* cells and expression clone was placed in the Gene pulser and a short pulse was applied. Immediately 800 µL LB was added to cuvette. Electroporated *Agrobacterium* cells was transferred to a microcentrifuge tube incubated in shaker at 28 °C for 3 h. 200 µL aliquot was spread an on a LB plate containing 10 µg ml^− 1^ rifampicin, 10 µg ml^− 1^ gentamycin, and 50 µg ml^− 1^ kanamycin and positive transformed colonies were selected. Colony-PCR was conducted to cross-validate the successful transformation.

*A. thaliana* Col-0 wild type plants were transformed with *Agrobacterium* cells carrying the 35 S::AT5g03350 CDS-pMDC32 expression clone (Additional File 3: Fig S2 E) using floral-dip method as described earlier [43]. Antibiotic selection of transformed plants was done using modified method of Harrison et al. (2006) [44]. The collected seeds of floral-dipped plants were surface sterilized with 5% calcium hypochlorite for 10 min and spread on ½ MS plates having 1% sucrose and 0.8% plant agar with 10 µg ml^− 1^ hygromycin (B) The plates were then incubated in 4 °C for stratification and synchronized germination. After 48 h of stratification, the plates were placed in plant growth chamber (Conviron, Adaptis 1000) at 23 °C, 150 µmol photons m^− 2^ s^− 1^ for 10–12 h and then incubated in dark at 23 °C for 3 days. The plates are then transferred to plant growth chamber at 23 °C temperature, 150 µmol photons m^− 2^s^− 1^, 60% relative humidity and 16 h light/ 8 h dark cycle. After another 2–3 days of growth, the hygromycin resistant seedlings were distinguished from the susceptible ones by longer hypocotyls and greener cotyledons (Additional File 6: Fig. S3A). Following that, the selected seedlings were transferred to ½ MS plates with 1% sucrose and 0.8% plant agar and grown normally in plant growth chamber for another 14 days. The seedlings with true leaves were then transferred to potted soil and grown for next generation seed collection. Transformed T_0_ plants were PCR verified using hygromycin phosphotransferase (*hpt*) gene specific primers (Additional File 6: Fig. S3A). Genomic DNA form each T_0_ plant was isolated using modified protocol of Murray et al. (1980) [45]. For copy number identification and homozygosity test in T_1_, T_2_, and T_3_ generation, the modified method of Kihara et al. (2006) was used [46]. Amplification of endogenous single-copy gene 4-hydroxyphenylpyruvate dioxygenase (*4hppd*) is used as a reference against the amplification of *hpt* gene. Briefly, PCR amplification of both the genes were done separately using DreamTaq PCR kit (DreamTaq, Thermo scientific) as per the manufacturer’s instrcutions from genomic DNA of each plant with amplification cycle of 28 in a 25 µl of reaction volume with same concentration of primers and templates (~ 150 ng DNA). 12 µl of each reaction was run in 1.5% (w/v) agarose gel (UltraPure™ Agarose, Invitrogen, 16500-500) and image was captured using chemidoc (ImageQuant LAS 500). Band intensity was quantified using ImageJ software (https://imagej.nih.gov/ij/download.html). Band intensity ratio of 1:1 between HPT and 4HPPD amplification for a line in T_1_, T_2_, and T_3_ generation confirms homozygosity with single insertion, 0.5:1 ratio confirms heterozygosity with single insertion and 2:1 suggests homozygosity with double insertion (Additional File 6: Fig S3 B, C, and D) For the following experiments, seeds of T_3_ homozygous single insertion line1 was used.

### Screening of At5g03350 gene specific SALK_036814 T-DNA insertion line

AT5G03350 locus specific t-DNA insertion lines, N536814 (SALK_036814) were procured from the Nottingham Arabidopsis Stock Centre (NASC) (http://arabidopsis.info/). The seeds were surface sterilized with 5% calcium hypochlorite for 10 min and spread on ½ MS plates with 1% sucrose and 0.8% plant agar. The plates were then incubated at 4 °C for stratification and synchronized germination. After 72 h of stratification, the plates were placed in plant growth chamber (Conviron, Adaptis 1000) at 23 °C, 150 µmol photons m^− 2^ s^− 1^, 60% relative humidity, and 16 h light/8 h dark cycle. 14 days old seedlings were transferred to soil and grown for another 7 days. The 21 days old plant leaves were used for genomic DNA isolation using modified cTAB method [45]. The primers for gene specific amplification (SALK_036814.56.00.x forward and reverse) were designed by T-DNA primer design tool (http://signal.salk.edu/tdnaprimers.2.html) and the LBP1.3 was used for T-DNA plus genomic DNA hybrid amplification (Additional File 2: Table S1). Predicted product length of PCR amplification of genomic DNA of this mutant line with SALK_036814.56.00.x forward and reverse primers was 1190 bp whereas, in wild type or heterozygous mutant plants LBP1.3 and SALK_036814.56.00.x RP primer specific product size was 598–898 bp. We have set two paired reactions for each genotypic study because of difference in Tm of LBP1.3 and gene specific primers (Additional File 7: Fig. S4).

### Confirmation of At5g03350 overexpression and At5g03350 mutant (SALK_036814) lines using qRT-PCR

To confirm the 35 S::At5g03350 overexpression and At5g03350 mutant lines, the transcript levels of the At5g03350 gene were examined and compared with Col-wild type plants using qRT-PCR, as described in the section on expression analysis of *AtLLPs* under six selected abiotic stresses.

### Detection of ROS production in At5g03350 mutant, At5g03350 overexpression and wild type plants under six chosen abiotic stresses

The selected abiotic stresses, cold, high light, oxidative, UV-B, wound, and ozone are known to enhance the production of ROS, such as singlet oxygen (^1^O_2_), superoxide radical (O_2_^•−^), hydrogen peroxide (H_2_O_2_), and hydroxyl radical (OH^•^) in plants [47–51]. To assess differences in the abiotic stress response of At5g03350 mutant (SALK_036814), At5g03350 overexpression, and wild type plants under the aforesaid abiotic stresses, the presence of two classical stress markers i.e. superoxide radical and hydrogen peroxide were detected using nitroblue tetrazolium (NBT) and 3, 3’diaminobenzidine (DAB), respectively were monitored in the control and stress exposed seedlings at selected time points, 0 (control), 2, 6, and 12 h as previously described by Mondal et al. (2021) [4].

### Statistical analysis

Data were expressed as mean ± standard deviation (SD) of at least three biological replicates. The results of expression data were statistically examined using one-way analysis of variance (ANOVA), followed by Student–Newman–Keuls test using SigmaPlot 12 (*p* < 0.05) to determine significant differences in control- and stress-treated samples [52].

## Results

### Chromosomal localization, gene structure, protein motif, peptide sequence, and phylogeny analyses

**Fig. 1:**
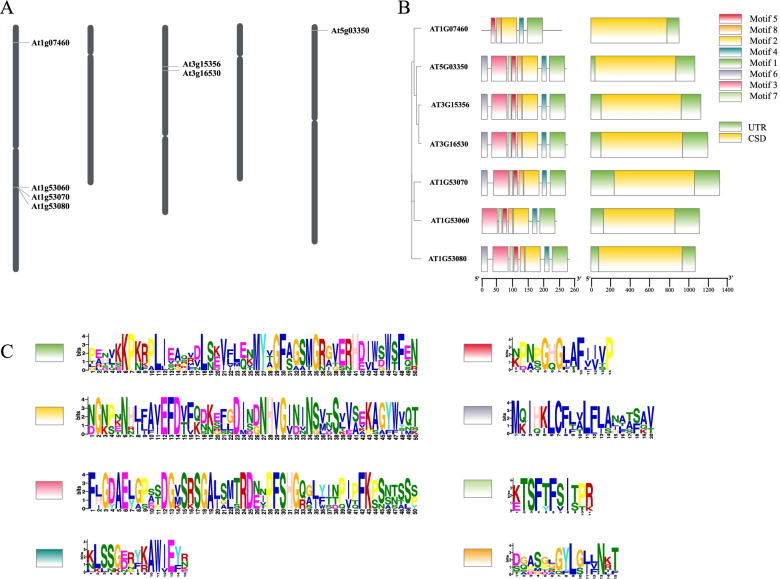
Chromosomal localization, sequence analysis, and phylogenetic relationship of *LLPs* in *Arabidopsis thaliana* (*AtLLPs*) (**A**) Chromosomal localization, (**B**) Phylogeny, gene, and protein structure, and (**C**) Web-Logo analysis of amino acid variability and conservation present in the identified conserved motifs of *AtLLPs*. The height of the stack is proportional to the conservation of the representative residue. Conserved motifs are shown in different colour boxes as depicted in the protein structure

Chromosomal localization investigation revealed that out of seven, four *AtLLPs*, AT1g53060, AT1g53070, AT1g07460, and AT1g53080, are located on chromosome 1 while two others, AT3g16530 and AT3g15356 are located on chromosomes 3 and one last, AT5g03350 is found on chromosome number 5 alone (Fig. 1 A). The localization study suggested that the AT1g53060, AT1g53070, and AT1g53080 genes on chromosome 1 were tandem-duplicated. Gene structure analysis demonstrated that all *AtLLPs* have a similar basic gene structure and are intronless (Fig. 1B). Although there is considerable variation in both the 5′ and 3′ UTR structure, this indicates that they are more flexible in terms of their functional diversity (Fig. 1B). Motif analysis revealed the presence of eight conserved motifs in most of the AtLLPs, all of which are arranged in the same order (Fig. 1B, C). The sequence alignments of peptide sequences in AtLLPs showed that lectin domains are substantially conserved while signal peptides (SP) are less conserved (Additional File 8: Fig. S5). AT1g53060 and AT1g07460 lack SP, whereas the other five AtLLPs include SP of varying lengths that are partially similar in sequence (Additional File 8: Fig. S5).

**Fig. 2:**
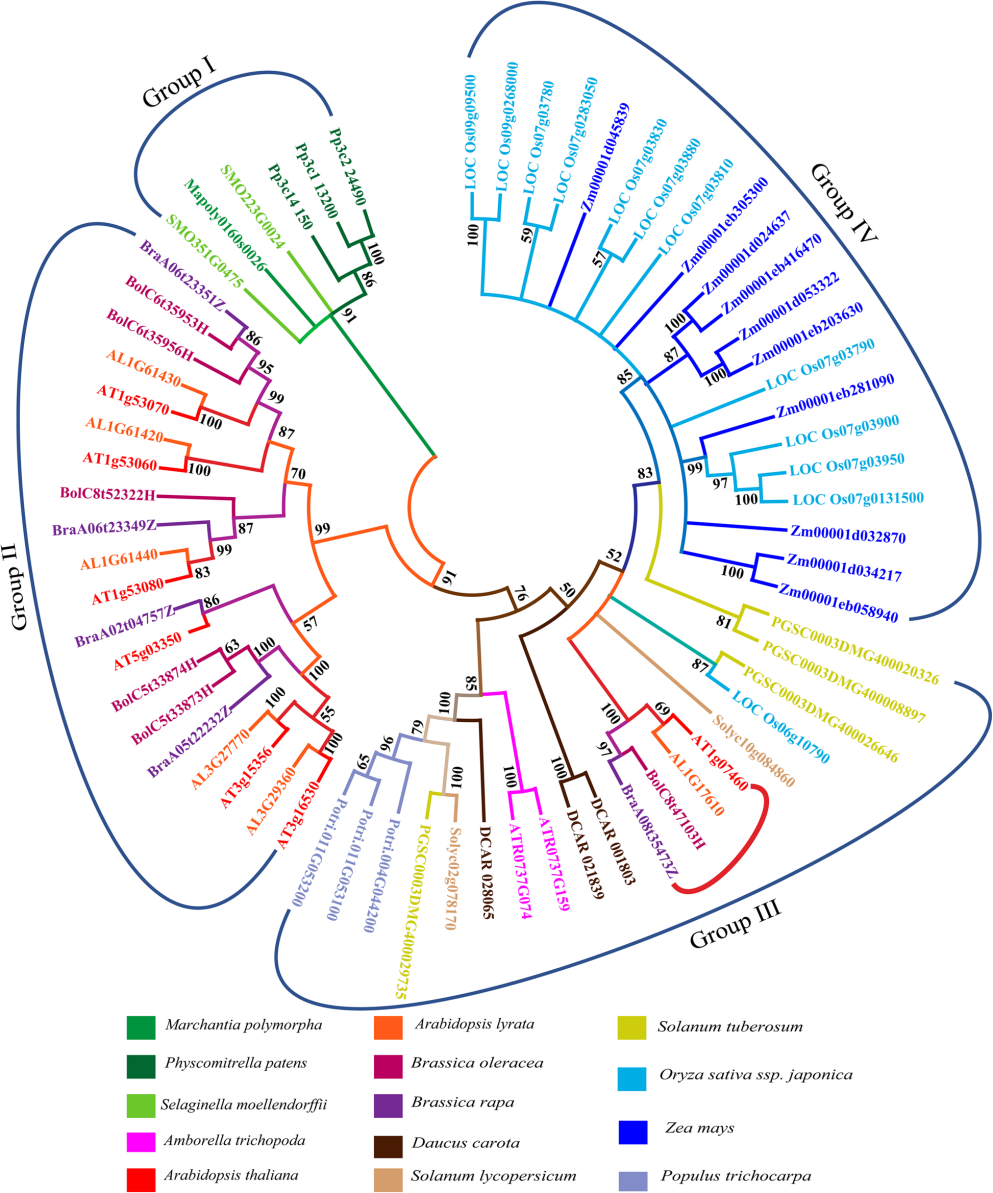
Phylogenetic relationship of LLPs in *Arabidopsis thaliana* (AtLLPs) and their orthologs present in *Marchantia polymorpha, Physcomitrella patens, Selaginella moellendorffii, Amborella trichopoda, Arabidopsis lyrata, Brassica oleracea, Brassica rapa, Daucus carota, Solanum lycopersicum, Solanum tuberosum, Oryza sativa ssp. japonica, Zea mays, Triticum aestivum*, and *Populus trichocarpa.* To construct the phylogenetic tree, the peptide sequences of AtLLPs and their orthologs were retrieved from NCBI and Phytozome. Peptide sequences were aligned using ClustalW program in MEGA 11. The neighbour-joining tree was constructed using MEGA 11 where bootstrap method used as a test of phylogeny. Note that 1000 bootstrap replicates were used in the test and bootstrap-values are presented as percentage (%) below each branch point. Orthologs from each species are color coded as represented by colored boxes below the tree. Group I is represented by bryophytes and pteridophytes, Group II by members of Brassicaceae, Group III by dicots, and Group IV by monocots

To understand the evolutionary relationship of AtLLPs with other plant groups, phylogenetic analysis was conducted and presented in the form of phylogenetic tree (Fig. 2). In the phylogenetic tree, lower non-seed plants made Group I, LLPs of Brassicaceae made a separate group as Group II, dicots made Group III, and monocots on Group IV (Fig. 2). Interestingly, AtLLPs are closely related to the non-seed group of plants rather than other dicots (Fig. 2). Although most of the LLPs are found to be closely related to non-seed plants, AT1g07460 and its orthlogs from members of other Brassicaceae species made a small group in Group III (highlighted in red bracket) and are more closely related to monocots (Fig. 2).

To have an improved understanding of AtLLPs, the *in silico* physicochemical analysis was conducted (Additional File 9: Table S2). With the exception of AT1g53060 and AT1g07460, which has a length of 242 and 258 amino acids (AA) respectively, AtLLPs are stable and have a length of 272–283 AA (Additional File 9: Table S2). AtLLPs have aliphatic indexes ranging from 69.26 to 80.50; however, their grand average hydropathicity is extremely diverse (Additional File 9: Table S2). AT3g16530 is nearly neutral in charge with a pI of 6.98 and AT1g07460 is acidic in nature with pI of 4.80, whereas the others are basic in nature.

### Sub-cellular localization prediction suggested AtLLPs could be located in intracellular compartments

Based on the SUBAcon algorithm, subcellular localization analysis of AtLLPs revealed that all investigated AtLLPs, with the exception of AT1g53060 and AT1g07460, are found in the extracellular spaces (Additional File 10: Table S3). This may be due to a lack of signal peptide in AT1g53060 and AT1g07460, as shown in Additional File 8: Fig. S5. Moreover, the locations of different AtLLPs have been predicted in different organelles such as, nucleus, mitochondria, and plastid (Additional File 10: Table S3). The diversity in the locations of AtLLPs shows the versatility in their functions.

### Evolutionary divergence analysis of *AtLLPs* gene family and its orthologs

The evolutionary divergence of six *AtLLPs* gene family with seven additional model plant species, including *Arabidopsis lyrata, Brassica rapa, Solanum lycopersicum, Zea mays, Selaginella moellendorffii, Physcomitrium patens*, and *Chlamydomonas reinhardtii*, was analyzed. The dN/dS ratios of *LLP* genes and their orthologous pairs were calculated to detect evolutionary pressure acting on these genes (Additional File 11: Table S4) according to Kryazhimskiy and Plotkint, (2008) [53]. A total 42 orthologs of six *AtLLP* genes were found from *Arabidopsis lyrata, Brassica rapa, Solanum lycopersicum, Zea mays, Selaginella moellendorffii, Physcomitrium patens*, and *Chlamydomonas reinhardtii. Chlamydomonas reinhardtii* showed one orthologous gene in its genome. The other two, *Physcomitrium patens* and *Selaginella moellendorffii* showed three and four orthologs, respectively. In higher plant species such as *Arabidopsis lyrata, Brassica rapa, Solanum lycopersicum, Zea mays* 5, 4, 6 and 5 orthologous genes were found, respectively.

For evolutionary pressure quantification, we estimated values of dN/dS ratios of the gene pairs and this was followed by estimation of evolutionary divergence time (Table 1, Additional File 11: Table S4). The dN/dS ratio of the LLP gene orthologs from Angiospermic members was found to be in the range of 0.0109–0.4634, whereas the ratio values ranged 0.0114–0.0463 when compared with lower plant species, *Selaginella moellendorffii, Physcomitrium patens and Chlamydomonas reinhardtii* (Additional File 11: Table S4). This analysis shows that the values of dN/dS ratio falling less than 1 (Additional File 11: Table S4), suggesting that *LLP* genes are under purifying (negative) selection, i.e. natural selection here suppresses protein changes.

**Table 1:** Estimated divergence time of *Arabidopsis thaliana LLP* gene family and its orthologs in *Arabidopsis lyrata, Brassica rapa, Solanum lycopersicum, Zea mays, Selaginella moellendorffii, Physcomitrium patens*, and *Chlamydomonas reinhardtii*

S. N.	Pair of plant Species	LLP gene divergence time (MYA*)	Molecular speciation time (MYA*)
1	* A. thaliana - A. lyrata*	0.3–1.7	4.7–10.2
2	* A. thaliana - B. rapa*	1.3–3.2	23.4–33.5
3	* A. thaliana - S. lycopersicum*	16–18	111–131
4	* A. thaliana - Z. mays*	16–18	115–308
5	* A. thaliana - S. moellendorffii*	63–182	410 – 468
6	* A .thaliana - P. patens*	51–173	465 – 533
7	* A. thaliana - C. reinhardtii*	190–208	970–1244

* MYA- Million years ago

### *In silico* expression analysis revealed that *AtLLPs* differently express under various abiotic stresses

**Fig. 3:**
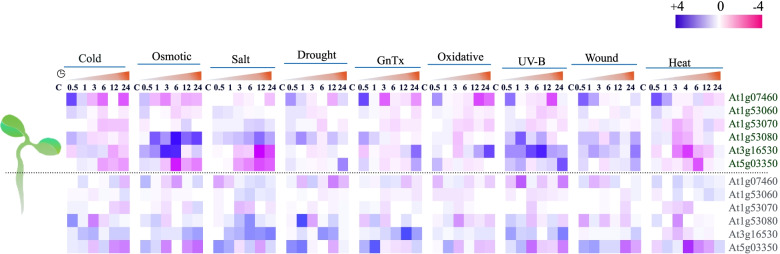
Temporal and tissue specific *in silico* expression analysis of *LLPs* in *Arabidopsis thaliana* (*AtLLPs*) under cold, osmotic, salt, drought, genotoxic, oxidative, UV-B, wound, and heat stress. A heat map was created using TBtools software version 0.665 (https://github.com/CJ-Chen/TBtools). Violet color in heatmap indicates up (+ 4) and pink color in indicates down-expression (-4) pattern of *AtLLPs* in different time points (C, control; 0.5, 1, 3, 6, 12, and 24 h) under the above mentioned abiotic stress. The color intensity is proportional to the absolute value of the log2-transformed ratios. A single dashed line was used to illustrate shoot and root specific differential expression pattern of *AtLLPs* during seedlings stage

Based on publicly available microarray datasets, *AtLLPs* were subjected to *in silico* expression analysis under various abiotic stresses, including cold, osmotic, salt, drought, genotoxic, oxidative, UV-B, wound, and heat stress (Fig. 3). The comprehensive analysis of expression profile from the microarray datasets demonstrated that a majority of tested *AtLLPs* expressed at high/intermediate levels in both root and shoot tissues under studied abiotic stresses (Fig. 3). As per this differential expression pattern, *AtLLPs* may be regulated by abiotic stresses and implicated in stress management.

### The promoter region of *AtLLPs* contains CREs involved in growth and development, hormone, and multi-stress response

**Fig. 4:**
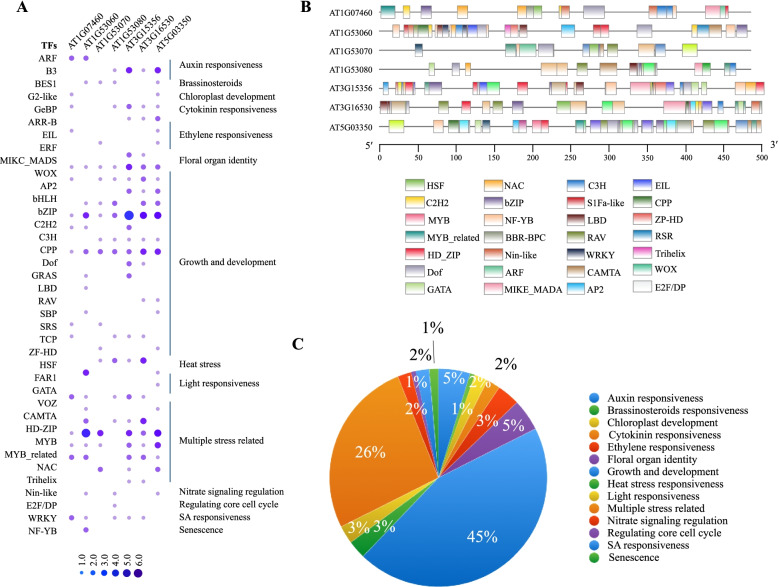
Promoter structure analysis and distribution of stress-associated potential TFs in the 500 bp upstream sequences of *LLPs* in *Arabidopsis thaliana* (*AtLLPs*). **A** Dot plot analysis with functional annotation of associated TFs those are available from PlantRegMap database http://plantregmap.gao-lab.org/. **B** Different colored boxes across the promoter length of *AtLLPs* represent the physical localization of identified TFs binding site (TFBSs). The colored box with corresponding TFs name was mentioned for batter understanding. **C** Proportional distribution pattern (in percentage) of functionally annotated growth-development and multiple stresses related TFs are represented in the pie chart

To understand transcriptional regulation of seven *AtLLPs* under various abiotic stresses, we analyzed the abundance of potential CREs using 500 bp upstream sequences of these genes (Fig. 4). The promoter structure analysis suggests that wide classes of CREs spanned across the 500 bp *AtLLPs* promoter. Dot plot analysis with functional annotation revealed that ~ 44% of CREs were involved in growth and development including WOX, AP2, bHLH, bZIP, C2H2, C3H, CPP, Dof, GRAS, LBD, RAV, SBP, SRS, TCP, and ZF-HD and ~ 27% modulate multiple stress (VOZ, CAMTA, HD-ZIP, MYB, MYB related, and NAC) (Fig. 4 A). Furthermore, different CREs regulated by plant hormones such as auxin (ARF and B3), cytokinin (GeBP), ethylene (EIL and ERF), salicylic acid (WRKY), and brassinosteroids (BES1) and certain important TFs regulates underlying response to light (FAR1, GATA), heat (HSF), cell cycle (E2F/DP), nitrate signaling (Nin-like), and identity of floral organ (MIKC_MADS) were also identified. Additionally, we have predicted the physical localization of motif of CREs across the promoter length (Fig. 4B). The proportional distribution pattern of different identified CREs and their functional annotations are represented in the pie chart (Fig. 4 C).

### Expressional correlation coefficients analysis between *AtLLPs* and their CREs cognate TFs supported the role of *AtLLPs* in different abiotic stress conditions

**Fig. 5:**
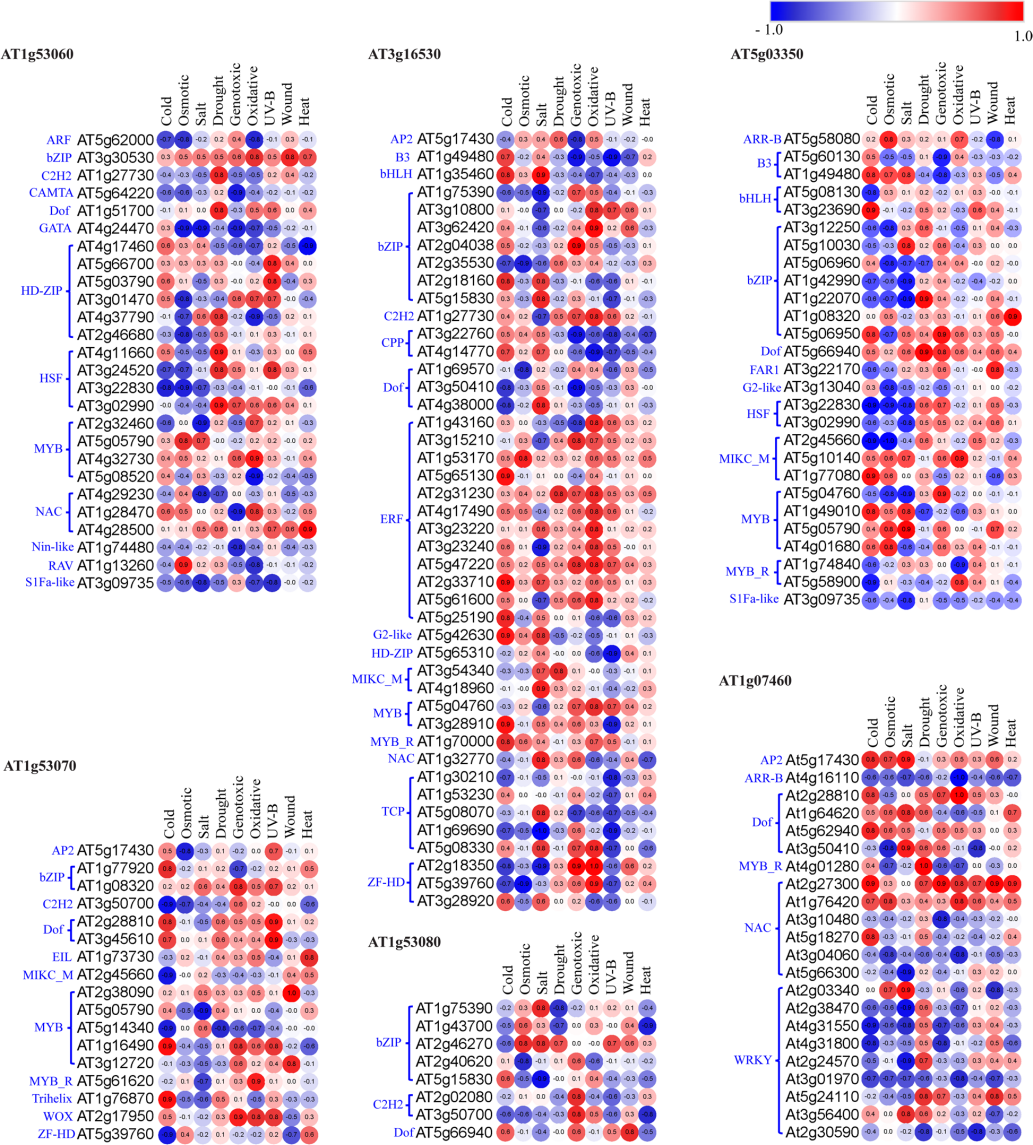
Heatmap showing the expressional correlation between *LLPs* in *Arabidopsis thaliana* (*AtLLPs*) and their CREs specific transcription factors (TFs), belonging to different TFs families (mentioned in blue color on left side of each heatmap) in the shoot tissues. Pearson correlation coefficient (r) was used to assess the pairwise expression similarity between the candidate *AtLLP* genes and their CREs cognate TFs during cold, osmotic, salt, drought, genotoxic, oxidative, UV-B, wound, and heat stress. Heatmap was created using Morpheus (https://software.broadinstitute.org/morpheus, version 1.0). Red represents positive and blue represents negative expression correlation. A scale showing critical values of r is shown with corresponding colors. Values with ± 0.8 (*p* < 0.05) was considered as a significant expression

**Fig. 6:**
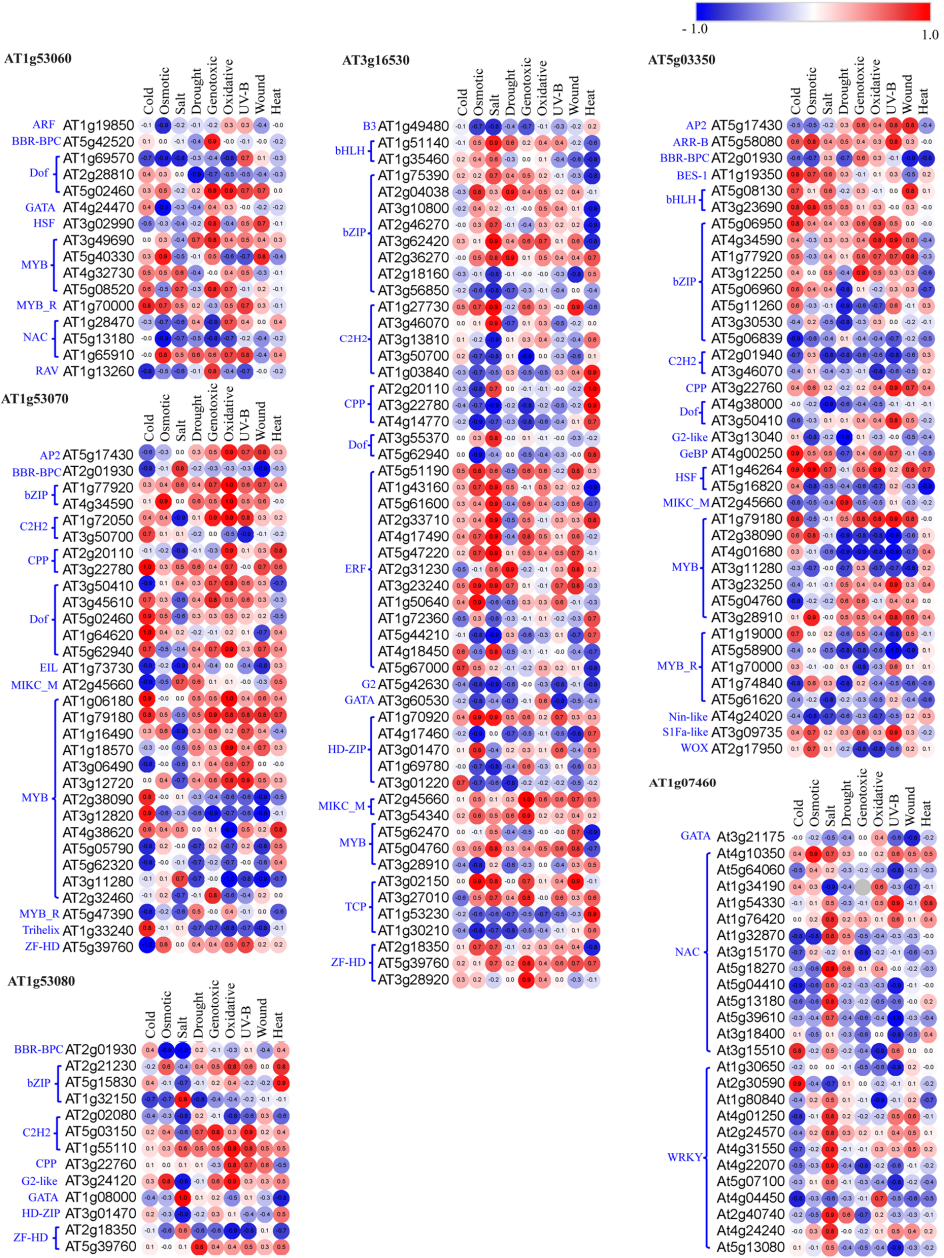
Heatmap showing the expressional correlation between *LLPs* in *Arabidopsis thaliana* (*AtLLPs*) and their CREs specific transcription factors (TFs), belonging to different TFs families (mentioned in blue color on left side of each heatmap) in root tissues. Pearson correlation coefficient (r) was used to assess the pairwise expression similarity between the candidate *AtLLP* genes and their CREs cognate TFs during cold, osmotic, salt, drought, genotoxic, oxidative, UV-B, wound, and heat stress. Heatmap was created using Morpheus (https://software.broadinstitute.org/morpheus, version 1.0). Red represents positive and blue represents negative expression correlation. A scale showing critical values of r is depicted with the corresponding colors. Values with ± 0.8 (*p* < 0.05) was considered as a significant expression

To strengthen our CREs-based results and validate the role of *AtLLPs* in multiple abiotic stresses, we predicted the cognate TFs that can bind to the CREs present in 500 bp upstream region of TSS. Additional File 12: Table S5 lists the predicted cognate TFs from major families mentioned in the previous section that can bind to the identified CREs in each of the *AtLLPs*. Based on publicly available *in silico* expression data of different abiotic stress series, used for *in silico* expression profiling of *AtLLPs* (Fig. 3), we calculated the expressional correlation of coefficients (r-value) between *AtLLPs* and their CREs specific TFs (Additional File 13: Table S6), to identify probable TFs that regulate the expression of *AtLLPs* under different abiotic stresses in both root and shoot tissues. This analysis showed that under different abiotic stresses, the tested *AtLLPs* have a positive or negative correlation with their CREs cognate TFs in both root and shoot tissue (Additional File 13: Table S6). Among these TFs, only a few of them, including abiotic stress-responsive TFs, exhibited significant positive or negative expressional correlation [± 0.8 (*p* < 0.05)] (Additional File 14: Table S7). The TFs that have demonstrated a significant expressional correlation with *AtLLPs* at least in a single tested abiotic stress are presented in the form of heatmap in shoot and root tissues (Figs. 5 and 6). The analysis demonstrated that the primary regulatory TFs are different for roots and shoots and for different abiotic stresses. The presence of ~ 27% abiotic stress-responsive CREs in the promoter of tested *AtLLPs* and their significant expressional correlation with CREs cognate TFs suggested the tight regulation of *AtLLPs* under different abiotic stresses in both root and shoot tissue. These results suggest that *AtLLP* genes in *A. thaliana* may play various functions, including abiotic stress response.

### Transcript analysis confirmed that *AtLLPs* are responsive to various abiotic stresses

**Fig. 7:**
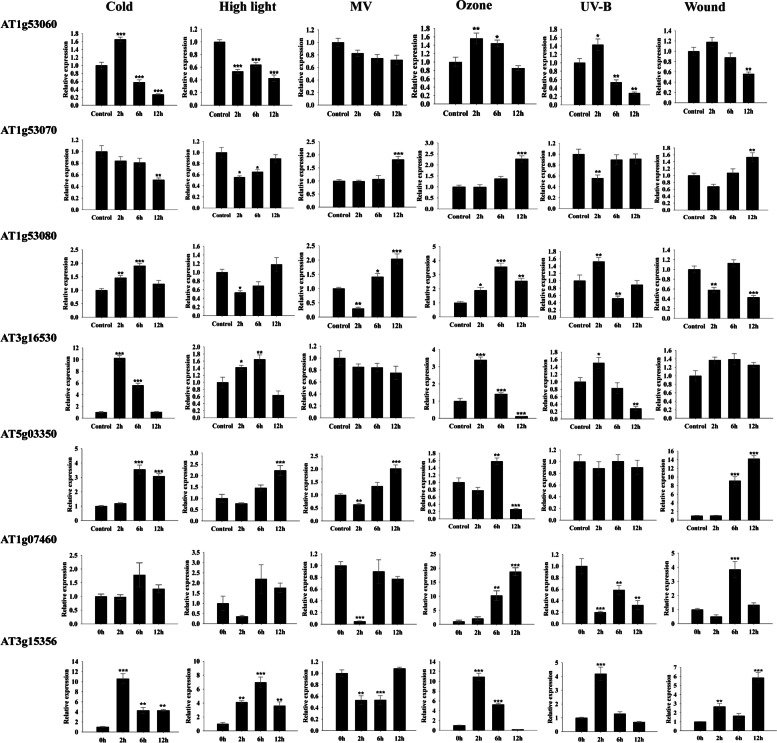
Quantitative-reverse transcription (qRT-PCR) based relative expression analysis of *LLPs* in *Arabidopsis thaliana* (*AtLLPs*) after 2, 6, and 12 h of exposure to six abiotic stresses such as cold, light, oxidative (MV), UV-B, wound, and ozone stress. Relative changes in expression level were normalized with two reference genes i.e., *AtAPT1* and *AtUBQ5*. Data represents the mean of fold increase over control sample ± SD of three biological replicates (*n* = 3). Single asterisk (*) indicates *p* < 0.05, double asterisk (**) indicates *p* < 0.01, and triple asterisk (***) indicates *p* < 0.001 for one-way analysis of variance (ANOVA), followed by Student-Newman-Keuls test performed among treated and control samples

Six abiotic stresses, including cold, high light, oxidative, UV-B, wound, and ozone stress, were selected to examine the temporal gene expression of seven *AtLLPs*. Most *AtLLPs* exhibited differential expression in all the tested stresses at 2, 6, and 12 h of exposure compared to control (Fig. 7; Additional File 15: Table S8).

The relative expression of AT1g53060 was significantly upregulated by > 1.5 fold at 2 h of cold, ozone, and UV-B. However, after 6 h of treatment, the expression was downregulated and reached to 0.27-, 0.85-, and 0.27-fold in cold, ozone, and UV-B treatment, respectively. For MV (oxidative) treatment, the expressional changes of this gene at all tested time points were insignificant. Moreover, AT1g53060 demonstrated significant downregulation under high light stress condition with the relative expression of 0.53, 0.63, and 0.42 fold at 2, 6, and 12 h of treatment, respectively. Furthermore, after wounding, the relative expression of this gene was insignificantly downregulated for the first 6 h; however, after 12 h of treatment, it showed a significant downregulation of 0.56-fold.

For AT1g53070, it shows a common pattern of expression under MV, ozone, and wounding. Changes in the relative expression of AT1g53070 was insignificant after 2 and 6 h of treatment; however, after 12 h of treatment, the gene expression was significantly upregulated to 1.81-, 2.73- and 1.53-fold in MV, ozone, and wounding, respectively. UV-B and high light stress initially downregulated the expression of this gene but reached a control level after 12 h of treatment. Under UV-B, the relative expression of AT1g53070 was 0.55-fold at 2 h; however, after 6 h, it reached the control level and remained the same till 12 h of treatment. Moreover, under high light, the relative expression of this gene was 0.55- and 0.64-fold at 2 and 6 h, respectively, and reached the control level after 12 h of treatment. Unlike the abovementioned expression patterns, this gene shows continuous downregulation under cold stress where the relative expression reached 0.51-fold after 12 h of treatment.

The relative expression of AT1g53080 and AT3g16530 did not show any distinct patterns under the studied abiotic stresses. Although after 12 h of MV exposure, AT1g53080 demonstrated significant upregulation (2.0-fold) in its expression. Furthermore, AT3g16530 showed a substantial increase in expression of 10.0- and 6.0-fold after 2 and 6 h of cold exposure, respectively. No significant and consistent changes of expression were observed for these two genes under other tested conditions.

Unlike these genes, AT5g03350 demonstrated the highest expressional upregulation after 6 and 12 h of treatments for most stresses. UV-B treatment did not significantly change the gene expression of this gene. Moreover, under ozone stress, 1.57 fold of expression was noticed at 6 h of treatment and then the declined expression reached to 0.26 fold after 12 h of treatment. The expression pattern of this gene was demonstrated to be similar under light, MV, and wounding. In these three stresses, the maximum increase of 2.22-, 2.01- and 14.19-fold were noticed after 12 h of stress treatment.

AT1g07460 did not display a clear pattern of expression during cold, high light, MV, and UV-B. However, under ozone and wounding stress, it demonstrated maximal expression of 18.71- and 3.83-fold after 12 and 6 h, respectively. The seventh gene in this family, AT3g15356, showed a substantial increase in expression of 10.57-, 6.96-, 10.91-, 4.19-, and 2.67-fold after 2 h of exposure to cold, high light, ozone, UV-B, and wounding, respectively. Its expression reduced as the duration of stress exposures increased, with the exception of wounding, when it rose 5.85-fold after 2 h.

The critical evaluation of the expression pattern of all the *AtLLPs* suggested that out of these seven, AT1g53070 and AT5g03350 are important candidates that may have a prominent role in abiotic stress response and related signaling pathways.

### At5g03350 mutant (SALK_036814) plants produced more ROS than wild type plants, whereas 35 S::At5g03350 overexpression plants produced less under six abiotic stresses

**Fig. 8:**
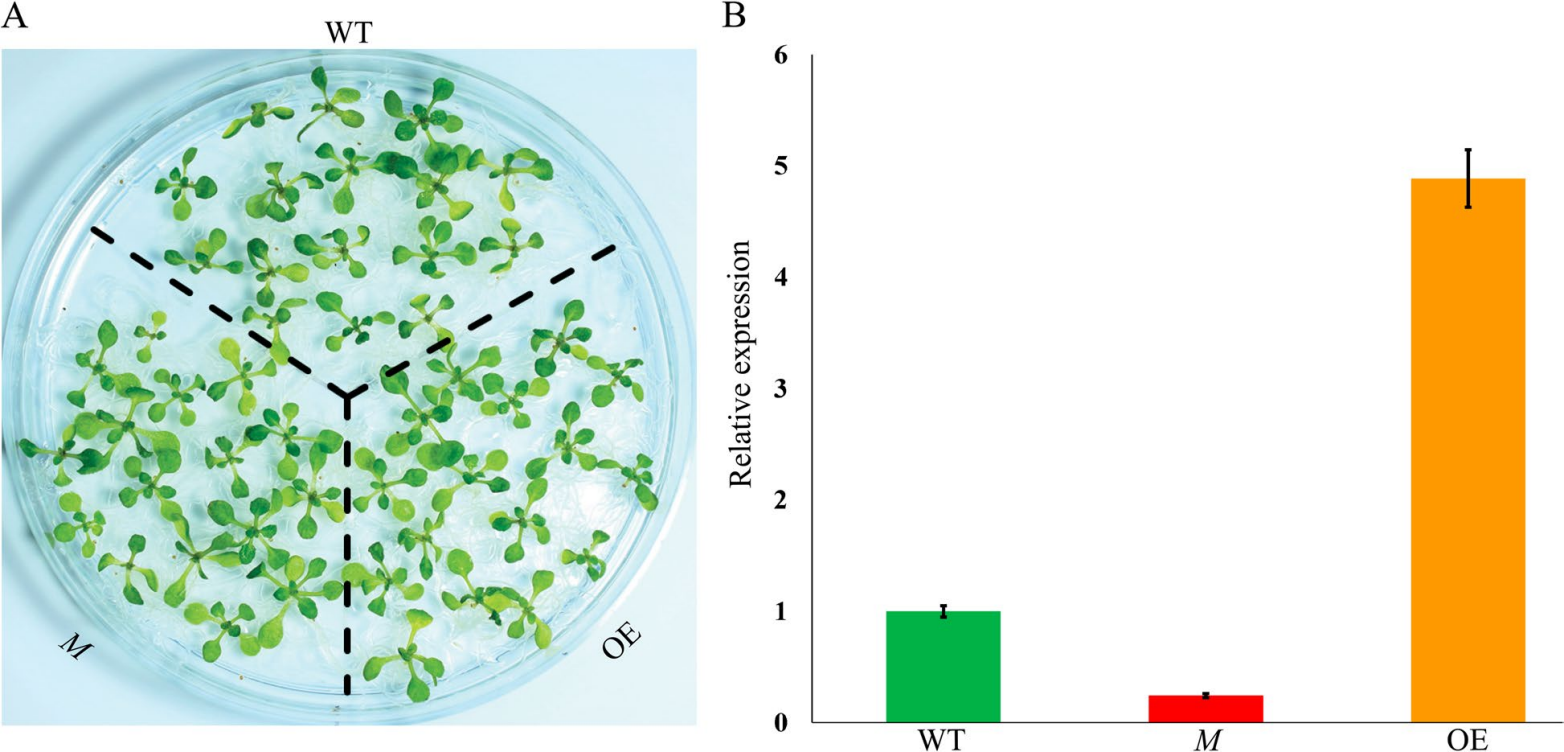
**A** The phenotypes of the 10-days-old wild type (WT), 35 S::At5g03350 overexpression (OE), and At5g03350: mutant (SALK_036814) plants (*M*) of *Arabidopsis thaliana* grown on ½ Murashige and Skoog (MS) medium plate (90 × 14 mm) with 1% sucrose and 0.8% plant agar. **B** Relative expression levels of At5g03350 gene in WT, OE, and *M* plants based on qRT-PCR analysis

At5g03350, one of the key genes under evaluated abiotic stresses among seven *AtLLPs* (Fig. 7; Additional File 15: Table S8), was selected for further functional characterization to validate *AtLLPs* involvement in abiotic stress tolerance. For that, ROS generation in terms of two stress indicators, superoxide radical (O_2_^•−^), hydrogen peroxide (H_2_O_2_), was examined in At5g03350: mutant (SALK_036814), 35 S::At5g03350 overexpression and Col-0 wild type plants subjected to the same six abiotic stresses, cold, high light, oxidative, UV-B, wound, and ozone, which were chosen for the transcript analysis of *AtLLPs* (Fig. 7; Additional File 15: Table S8). However, before examining abiotic stress-induced ROS generation in At5g03350: mutant (SALK_036814), 35 S::At5g03350 overexpression and Col-0 wild type plants, the expression levels of the At5g03350 gene were examined by qRT-PCR to evaluate relative expressional fold difference (Fig. 8). Data showed that the expression level of the At5g03350 gene was significantly reduced to ~ 4.0-fold in At5g03350 mutant plants, but significantly increased to ~ 5.0-fold in At5g03350 overexpression plants compared to wild type plants (Fig. 8). Moreover, expressional difference between mutant and overexpression plants is ~ 20 fold.

**Fig. 9:**
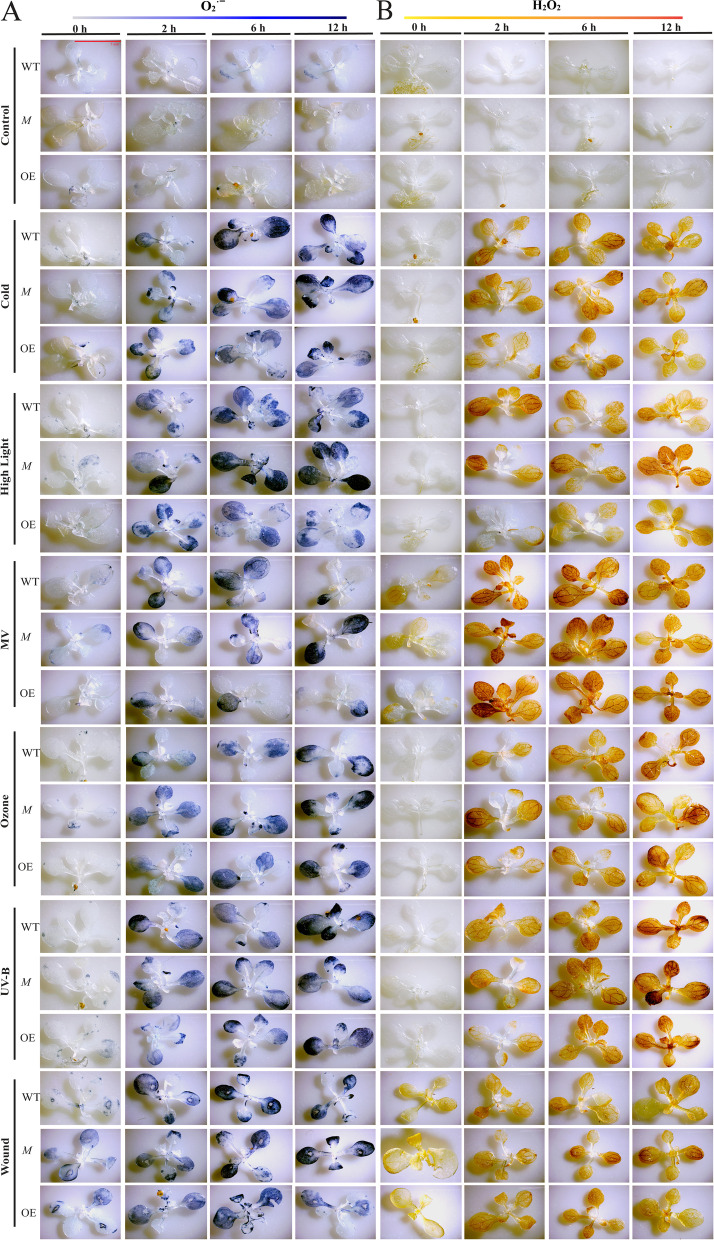
ROS accumulation in terms of two stress markers i.e. superoxide radical (O_2_^•−^), hydrogen peroxide (H_2_O_2_) was visualized using (**A**) nitroblue tetrazolium (NBT) and (**B**) 3,3- diaminobenzidine (DAB) staining, respectively, 10-days-old wild type (WT), 35 S::At5g03350 overexpression (OE), and At5g03350: mutant (SALK_036814) seedlings (*M*) of *Arabidopsis thaliana* following 0, 2, 6 and 12 h of treatments with six abiotic stresses, cold, high light, oxidative (MV), UV-B, wound, and ozone. At least three independent experiments were performed for each analysis and five seedlings of each genotype were examined. Bar = 5 mm

The production of superoxide radical (O_2_^•−^), hydrogen peroxide (H_2_O_2_) was detected using nitroblue tetrazolium (NBT) and 3,3- diaminobenzidine (DAB) staining, respectively in At5g03350: mutant (SALK_036814), 35 S::At5g03350 overexpression and Col-0 wild type plant seedlings after 0, 2, 6, and 12 h of aforesaid abiotic stresses (Fig. 9 A,B). Among the tested stresses, under high light, MV, UV-B, and wound, mutant seedlings showed higher stain colour intensity than wild type till the 12 h of treatment, indicating higher ROS accumulation, whereas overexpression seedlings showed lower stain colour intensity than wild type till the 12 h of treatment, indicating lower ROS accumulation (Fig. 9 A,B). In the case of cold and ozone stress, however, the observable differences in stain colour intensity are quite minimal (Fig. 9 A,B). According to these findings, At5g03350 gene has a protective role in *A*. *thaliana* against high light, MV, UV-B, and wound stress.

## Discussion

The transmission of environmental cues from the CW to the PM is an important aspect of stress perception, mitigation, and signaling. Apoplastic regions play an important function in information transmission. Environmental signals are received and recognized by the plant signaling pathways that may transmit these signals to the nucleus and finally result in the induction of stress-specific transcriptional and physiological responses. Plants have a large number of CW and PM receptors that detect environmental stimuli and initiate adaptive responses *via* downstream signal cascades. Among them, Lec-RLKs are known to be important for transmitting signals from the CW to the PM under stressful conditions. A group of Lec-RLKs, known as LLP, lack TM and kinase domains, indicating their full extracellular nature; moreover, they may act as a labile sensor/carrier for signal transmission from CW to PM. Because LLPs share structural similarity with the ectodomains of RLKs, which are well known to play a role for biotic and abiotic stresses [4, 54]. Therefore, it could be possible that the LLP subfamily of Lec-RLKs in *A. thaliana* may have certain role in abiotic stress mitigation/signaling. Therefore, the primary focus of this study is to examine the possible role of LLPs in *A*. *thaliana* (*AtLLPs*) under different abiotic stresses using *in silico* and wet lab experiments.

Since the molecular regulation and function of *AtLLPs* to multiple abiotic stresses is unclear, we investigated structural and functional aspects of *AtLLPs* in terms of chromosomal localization gene and protein structure, biochemical properties, phylogenetic relationship, sub-cellular localization, evolutionary divergence, *in silico* expression, promoter sequences, and transcript analyses under several abiotic stresses.

Most plant gene families are attributed to gene duplication either as an individual chromosomal duplication or segmental duplication in the same chromosome [55]. The chromosomal distribution patterns of *AtLLPs* show that the expansion of these genes in *A. thaliana* may have occurred because of simultaneous tandem gene duplications on chromosome 1 (Fig. 1 A). Such duplicated genes are considered to facilitate the rapid neo-functionalization that helps plants acclimate to harsh environmental conditions. Furthermore, the gene structure analysis of *AtLLPs* shows that introns are completely absent in them (Fig. 1B). The lack of introns may cause them to be less stable; however, it makes them capable of providing rapid functioning capabilities by avoiding splicing. Similar explanations have been proposed for other genes, in which intronless genes have been reported to have lower RNA stability [56]. Peptide sequence alignments and protein motif analyses demonstrated that *AtLLPs* have a highly conserved arrangement of motifs; however, their sequences are not similar (Fig. 1B,C, Additional File 8: Fig. S5). The signal peptides of these proteins were different in length and sequence (Additional File 8: Fig. S5). These results show a common mode of action for all *AtLLPs*, which might be spatially distant and distributed throughout the cell wall, intercellular space, apoplastic fluid, and other cellular organelles. This result is corroborated by sub-cellular localization analysis where *AtLLPs* are expected to be present in chloroplast, mitochondria, and nucleus (Additional File 10: Table S3).

Phylogenetic analysis revealed that AtLLPs except AT1g07460 are more closely related to their orthologs present in lower plants, i.e., bryophytes and pteridophytes rather than to other dicots and monocots (Fig. 2). The presence of orthologs of AtLLPs in early land plants such as bryophytes suggest that the related genes played an important role in combating against biotic and abiotic stresses. Subsequently, in the due course of evolution, functional diversification had taken place in these LLPs, which lead to the separation into dicot and monocots, which is nicely explained by the results of our phylogenetic analysis (Fig. 2).

Protein sequences and structures diverge from one another as they evolve from a common ancestor [57, 58]. The evolutionary rate ratio (dN/dS) is the oldest and most extensively used approach for determining selection pressure for protein-coding genes. Commonly, selective pressures on proteins are determined by comparing nucleotide sequences. Consequently, we compared the dN/dS ratios of *AtLLP* genes, as well as its orthologous pairs to examine the evolutionary divergences of the *AtLLPs* gene family over time (Additional File 11: Table S4, Table 1). As per evolutionary pressure analyses, AtLLPs are under negative selection, which indicates that natural selection suppresses protein modifications. Negative selection is effective for multiple housekeeping genes because mutations cause death or reduced fitness [59]. From this analysis, nature retained the roles of LLPs as housekeeping, indicating that they have been important for plant fitness/survival and stress mitigation/signaling from very early days.

Since abiotic stresses alter transcriptional regulation and activate stress-responsive genes for stress mitigation, the analysis of gene expression in response to stresses is an important and frequently investigated aspect. Therefore, we initiated the expressional analysis of *AtLLPs* using publicly available expression data (Fig. 3). Differential expression pattern of six selected *AtLLPs* by *in silico* expression profiling across a wide range of abiotic stresses, namely, cold, osmotic, salt, drought, genotoxicicty, oxidative, UV-B, wounding, and heat strengthened the idea of the abiotic stress regulation of AtLLPs and their role in abiotic stress and related signaling pathways.

CREs are short DNA sequences that TFs use to regulate gene expression [60]. Therefore, we performed structural analysis of *AtLLP* promoter sequences and CRE prediction for different TF binding sites (TFBS) (Fig. 4). Although computational identification and distribution of CREs do not confirm tissue-/time-specific regulatory mechanism, the presence of CREs provides an indication of the probable regulatory cues of gene expression. The promoter sequence analysis of *AtLLPs* identified multiple important consensus sequences under the functional categories of biotic and abiotic stress responsiveness, hormone signaling pathways, and development (Fig. 4). The presence of these abiotic stress-responsive CREs or TFBS in the promoter region of *AtLLPs* shows that the expression of *AtLLPs* may be influenced by multiple environmental signals such light, cold, and wounding.

Transcription is regulated by a number of proteins known as TFs, which form complexes involving protein–protein and protein–DNA interactions. Individual TFs bind to their cognate CREs or TFBS; therefore, we identified CREs cognate TFs in *AtLLPs* and analyzed the expressional correlation between tested *AtLLPs* and their CREs cognate TFs under cold, osmotic, salt, drought, genotoxic, oxidative, UV-B, wound, and heat stress (Figs. 5 and 6). We reported that that in each *AtLLPs*, the CREs cognate TFs differ but the expression of at least few of them was significantly correlated with their respective *AtLLPs* in each tested abiotic stresses in both root and shoot tissues (Figs. 5 and 6). This suggests that *AtLLPs* might be differentially regulated spatially and conditionally by the different sets of TFs under different abiotic stresses. Thus, as shown in Fig. 3, the results of expressional correlation analysis between tested *AtLLPs* and their CREs-specific TFs supported the differential expression patterns of *AtLLPs* under different abiotic stress conditions in both shoot and root tissues.

To experimentally verify the results of computational analysis, *A. thaliana* seedlings were subjected to abiotic stresses for 2, 6, and 12 h, and the expression profiling of seven *AtLLPs* was performed using qRT-PCR (Fig. 7).

The qRT-PCR result showed that the expression of AT1g53080 and AT3g16530 was inconsistent under the stress conditions that were tested (Fig. 7), suggesting they may not be an important player against abiotic stresses. The expression patterns of AT1g53060 were significantly downregulated in all tested stresses except MV (Fig. 7). Because AT1g53060 demonstrated initial upregulation in cold, ozone and UV-B stresses but significantly downregulated after 12 h except MV (Fig. 7), suggesting its role in abiotic early stress responses.

The AT1g07460 gene demonstrated maximal expression of 18.71- fold after 12 h in ozone and 3.83-fold after 6 h in wound stress (Fig. 7), indicating a possible role in these abiotic stresses. Further, another gene of this family, AT3g15356, showed a substantial increase in expression after 2 h of exposure to cold, high light, ozone, UV-B, and wound (Fig. 7), suggesting its role in early abiotic stress responses similar to AT1g53060.

However, the expression of other two genes, AT1g53070 and AT5g03350, was upregulated in most stresses tested and retained high transcript levels till 12 h of stress exposure (Fig. 7), suggesting their potential to be the important representative *AtLLP* genes under abiotic stresses (Fig. 7). The first key representative gene, AT1g53070, registered significant accumulations in its transcripts after exposures to MV and wound stress for 12 h (Fig. 7). Because these stresses are extensively known to increase reactive oxygen species (ROS) production in *A. thaliana* [49, 51, 61], the upregulation of its transcripts suggests that this gene may be regulated by ROS. The presence of an oxidative stress-induced CREs in the promoter region of this gene (Fig. 4) supports our results. The AT1g53070 expression was similarly up-regulated in response to ozone stress after 12 h (Fig. 7). The up-regulation of this gene can be supported by the fact that ozone induces a burst of ROS in apoplast and can induce extensive changes in gene expression [62]. Similar to this gene, the other members of RLK family have been demonstrated with their increased gene expression under ozone stress [4, 63]. Temperature is one of the most important environmental factors that influence plant growth and development. Plants are affected by both high and low temperatures, which disturbs their normal cellular machinery. Unlike other abiotic stresses, AT1g53070 transcript level was maintained till 6 h but dropped after 12 h of cold stress (Fig. 7), indicating its thermo-sensitive nature.

The expression of the second key representative *AtLLP*, AT5g03350 demonstrated significant change in all of the assessed abiotic stresses (Fig. 7). The up-regulation of its transcripts was detected during cold stress from 6 to 12 h (Fig. 7), and this change in expression could be supported by recent study which showed that Lec-RLKs have a role in cold stress in antarctic mosses [64, 65]. AT5g03350 transcripts accumulated the most after 6 or 12 h of exposure to high Light, MV, and ozone (Fig. 7), all of which are well-known oxidative stressors in plants [50, 66, 67]. This expression pattern shows that it, like AT1g53070, is regulated by ROS. Other Lec-RLKs have been shown to have ROS-induced expressions when exposed to a ROS-inducing stress [12, 68]. Similarly, AT5g03350 expression showed maximal expression after 12 h in response to the wound (Fig. 7). Another Lec-RLK was demonstrated to be up-regulated in *Populus nigra* in response to wounding, which supports our results [69].

These expression results suggest that out of seven, AT1g53070 and AT5g03350 genes may be involved in Arabidopsis responses to multiple environmental stresses. To support this hypothesis, AT5g03350 was chosen as a key representative for further functional characterization using its overexpression and mutant lines (Figs. 8 and 9). The visualization of ROS generation in wild type, At5g03350 overexpression, and At5g03350 mutant (SALK_036814), plants subjected to the same six abiotic stresses as in the transcript study (Fig. 7) revealed that At5g03350 mutant had higher ROS than wild type while At5g03350 overexpression had lower ROS under high light, MV, UV-B, and wound (Fig. 9). This finding shows the essentiality of At5g03350 gene in combating these abiotic stresses. The findings were further corroborated by our qRT-PCR data, which showed that the transcript level of At5g03350 gene increased after 12 h of exposure to these stresses (Fig. 7).

**Fig. 10:**
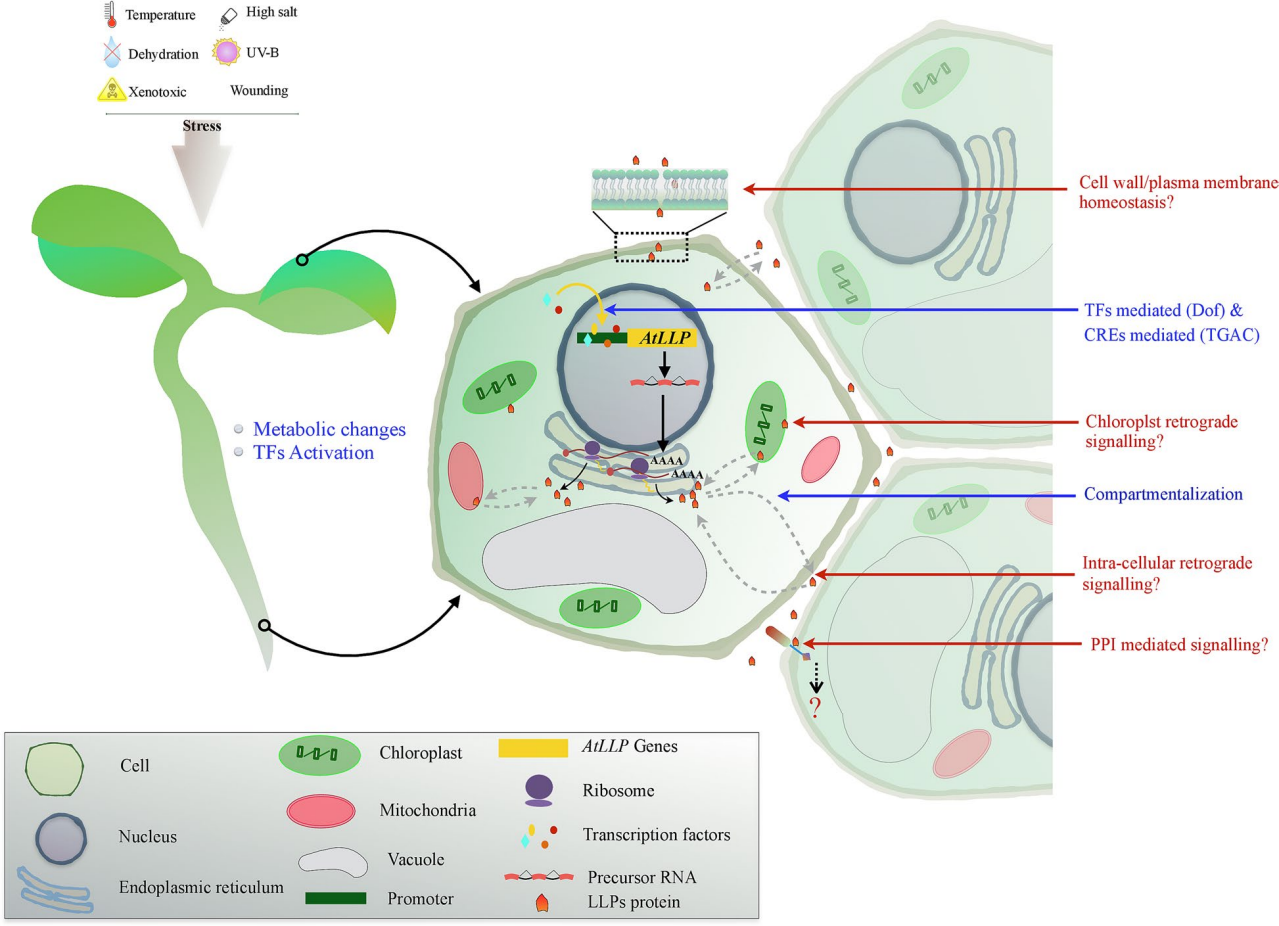
An overview of cellular responses and probable involvement of *LLPs* in *Arabidopsis thaliana* (*AtLLPs*) under various abiotic stresses. Present data mining bioinformatics and gene expression analyses suggested that in response to abiotic stress treatment such as cold, osmotic stress, drought, salinity, UV-B, heat, wound, and genotoxic (GnTx) compounds, *AtLLPs* are transcriptionally regulated. The activation of TFs of *AtLLPs* might be attributed to the alteration of cellular metabolic events in responses to abiotic stress. According to the results of a literature study, translated AtLLPs were compartmentalized in distinct organelles as well as the plasma membrane/cell wall, and the process was retrograded (dashed line arrows). AtLLPs may interact and activate downstream events in surrounding cells. Although the cascade of activation of downstream events till not elucidated. Blue-colored arrow indicates transcriptional and translational events, whereas red-colored arrows probable functions of AtLLPs. In the box, the name of the cellular organelles and molecules, which are illustrated in the model are mentioned

Based on the above results, the possible roles of AtLLPs in response to abiotic stresses in *A. thaliana* were discussed and summarized in the form of a hypothetical model (Fig. 10).

## Conclusions

Based on the results of this study, we propose that out of seven, two important representative *AtLLPs*, AT1g53070 and AT5g03350, have strong potential to play a role in abiotic stress mitigation and related signaling in *A. thaliana*. Additional investigation is required to establish the exact role of these genes in terms of ligands binding and signaling pathways in which they may be involved.

## Supplementary information

**Additional file 1: Fig. S1. **A denaturing formaldehyde agarose gel electrophoresis image for RNA.

**Additional file 2: Table S1. **List of primers used in the current study.

**Additional file 3: Fig. S2. **Represents the steps of overexpression line (At5g03350::overexpression) creation using gateway cloning technology followed by *Agrobacterium* mediated transformation.

**Additional file 4: **Sequencing information of AT5g03350CDS-pDONR207 entry clone (forward).

**Additional file 5:** Sequencing information of AT5g03350CDS-pDONR207 entry clone (reverse).

**Additional file 6: Fig. S3. **Represents the steps for PCR-based genotyping for At5g03350:: overexpression line.

**Additional file 7: Fig. S4.** Steps of PCR-based genotypic analysis of AT5g03350 gene-specific T-DNA insertion line.

**Additional file 8: Fig. S5.** Peptide sequence alignment of AtLLPs showing the presence of highly conserved lectin-binding domain and less conserved signal peptide.

**Additional file 9: Table S2. **Physicochemical properties of AtLLPs.

**Additional file 10: Table S3. **Predicted and experimentally confirmed subcellular location of *AtLLPs*.

**Additional file 11: Table S4. **Estimation of divergence time of *Arabidopsis thaliana LLP* gene family and its orthologs in *Arabidopsis lyrata*, *Brassica rapa*, *Solanum lycopersicum*, *Zea mays*, *Selaginella moellendorffii*, *Physcomitrium patens*, and *Chlamydomonas reinhardtii*.

**Additional file 12: Table S5.** List of transcription factors (TFs), which are predicted to bind *cis*-regulatory elements (CREs) present in 500 bp upstream promoter region of *AtLLPs*.

**Additional file 13: Table S6.** Expressional correlation of coefficients (r-value) analysis between *AtLLPs* and their *cis*-regulatory elements (CREs) cognate transcription factors (TFs) in root and shoot tissues under different abiotic stress conditions.

**Additional file 14: Table S7. **Number of predicted transcription factors (TFs), which can bind cognate *cis*-regulatory elements (CREs) in the promoter region of *AtLLPs*, as well as the list of TFs not included in this study and the number of TFs that are significantly correlated under different stress conditions in both root and shoot tissues.

**Additional file 15: Table S8. **Raw data of qRT-PCR.

## Data Availability

The datasets analyzed during this study are publicly available in Bio-Analytic Resource for Plant Biology (BAR; http://bar.utoronto.ca), TAIR (www.arabidopsis.org), SUBA4 (https://suba.plantenergy.uwa.edu.au/) and PlantRegMap (http://plantregmap.gao-lab.org/) repository.

## Abbreviations

CMV(Y): Cucumber mosaic virus
CREs: *Cis*-regulatory elements
gff: General feature format
LLPs: Legume lectin-like proteins
MV: Methyl viologen
PGR: Plant growth regulator
qRT PCR: Quantitative-reverse transcription polymerase chain reaction
SAI-LLP1: Salicylic acid induced-Legume Lectin like protein 1
SAR: Systemically acquired resistance
TAIR: The Arabidopsis information resource
TSS: Translation start site
UV-B: Ultraviolet-B rays
