# Supplementary material for: In silico characterization, molecular phylogeny, and expression profiling of genes encoding legume lectin-like proteins under various abiotic stresses in Arabidopsis thaliana
Source: BMC Genomics. 2022 Jun 29;23:480. doi: 10.1186/s12864-022-08708-0 (PMC9241310; doi:10.1186/s12864-022-08708-0)
Supplement: Supplementary file 1 — Additional file 1: Fig. S1. A denaturing formaldehyde agarose gel electrophoresis image for RNA. [file 12864_2022_8708_MOESM1_ESM.pptx]

## Slide 1
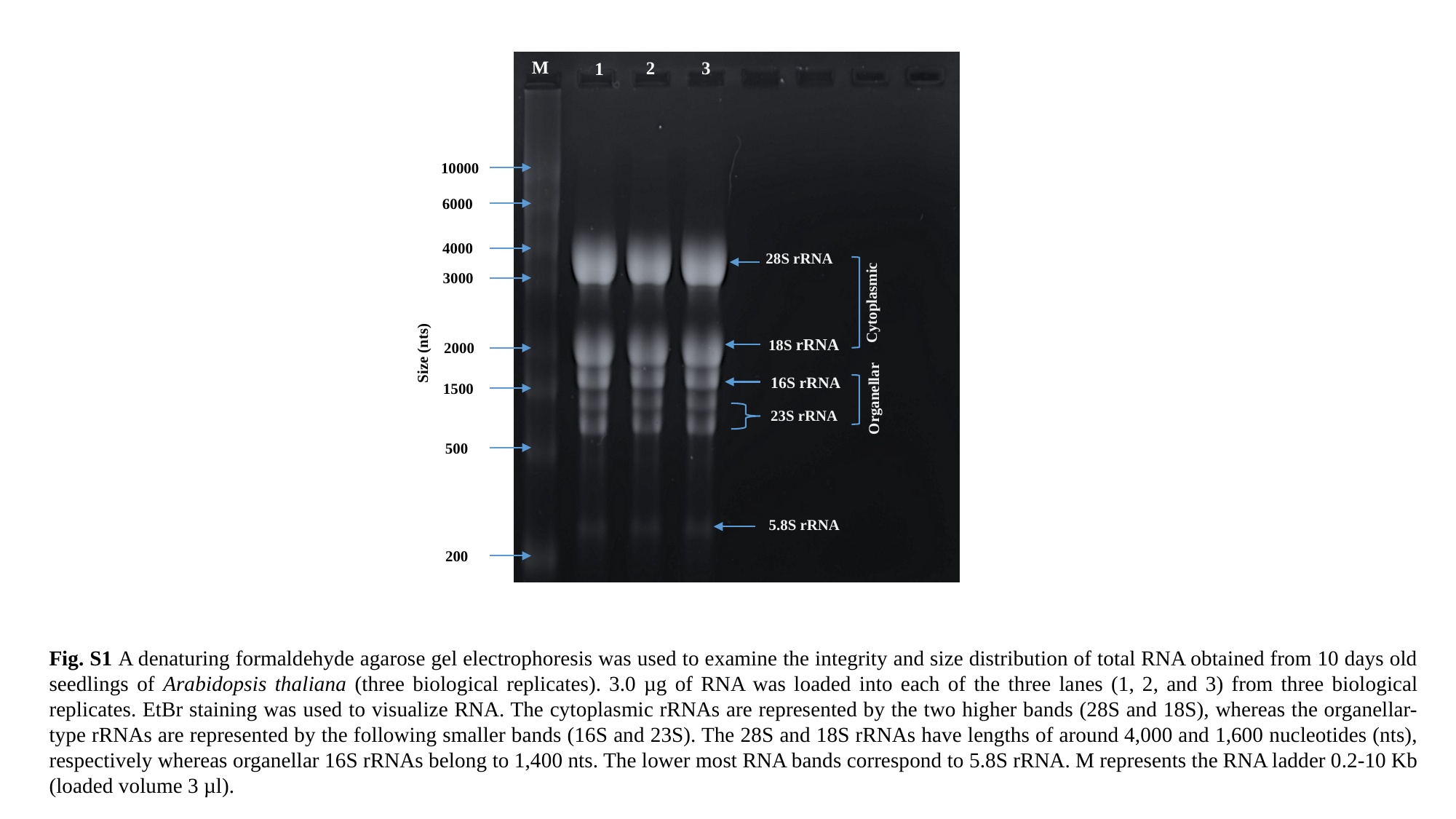

M
2
3
1
10000
6000
4000
3000
2000
1500
500
200
28S rRNA
18S rRNA
Organellar
5.8S rRNA
Cytoplasmic
Size (nts)
16S rRNA
23S rRNA
Fig. S1 A denaturing formaldehyde agarose gel electrophoresis was used to examine the integrity and size distribution of total RNA obtained from 10 days old seedlings of Arabidopsis thaliana (three biological replicates). 3.0 µg of RNA was loaded into each of the three lanes (1, 2, and 3) from three biological replicates. EtBr staining was used to visualize RNA. The cytoplasmic rRNAs are represented by the two higher bands (28S and 18S), whereas the organellar-type rRNAs are represented by the following smaller bands (16S and 23S). The 28S and 18S rRNAs have lengths of around 4,000 and 1,600 nucleotides (nts), respectively whereas organellar 16S rRNAs belong to 1,400 nts. The lower most RNA bands correspond to 5.8S rRNA. M represents the RNA ladder 0.2-10 Kb (loaded volume 3 µl).
