# Supplementary material for: In silico characterization, molecular phylogeny, and expression profiling of genes encoding legume lectin-like proteins under various abiotic stresses in Arabidopsis thaliana
Source: BMC Genomics. 2022 Jun 29;23:480. doi: 10.1186/s12864-022-08708-0 (PMC9241310; doi:10.1186/s12864-022-08708-0)
Supplement: Supplementary file 2 — Additional file 2: Table S1. List of primers used in the current study. [file 12864_2022_8708_MOESM2_ESM.docx]

| **S. N.** | **AGI/Gene name** | **Primer** | **Sequence** | **Purpose** |
| --- | --- | --- | --- | --- |
| **1** | AT1g53060 | Forward | ACGAGAACGGGAAAGTGACA | qRT-PCR |
|  |  | Reverse | ACCGGCAAAACCGACATACA | qRT-PCR |
| **2** | AT1g53070 | Forward | CGGTTTTGCTGGTTCGATGG | qRT-PCR |
|  |  | Reverse | GCACACCAGGGTAAACCAGA | qRT-PCR |
| **3** | AT1g53080 | Forward | TCATTGTTTGGCTTGCACCTG | qRT-PCR |
|  |  | Reverse | CGACCCATCGAACCGGAAAA | qRT-PCR |
| **4** | AT3g16530 | Forward | CCGGTTTGGTTCTGTTTATAGGC | qRT-PCR |
|  |  | Reverse | AATGAAACCACGCACTTGGC | qRT-PCR |
| **5** | AT5g03350 | Forward | GCCTTTGATCGTTGCCCATTT | qRT-PCR |
|  |  | Reverse | TGTTCTGGAAGGTCCAACTCC | qRT-PCR |
| **6** | AT1g07460 | Forward | GACCTTGATCCCATTCCAGA | qRT-PCR |
|  |  | Reverse | AAGACCAATGTGTTTGCCTC | qRT-PCR |
| **7** | AT3g15356 | Forward | TTTGGAGCTGGTCGTTTGAA | qRT-PCR |
|  |  | Reverse | ACAATGCAGAGTAAGCCACA | qRT-PCR |
| **8** | AtUBQ5 | Forward | CCAAGCCGAAGAAGATCAAG | qRT-PCR |
|  |  | Reverse | ACTCCTTCCTCAAACGCTGA | qRT-PCR |
| **9** | AtAPT1 | Forward | GAGACATTTTGCGTGGGATT | qRT-PCR |
|  |  | Reverse | CGGGGATTTTAAGTGGAACA | qRT-PCR |
| **10** | AT5g03350:OE | Forward | GGGGACAAGTTTGTACAAAAAAGCAGGCTTAATGAAGATTCATAAACTC | AT5g03350::CDS amplification |
|  | AT5g03350:OE | Reverse | GGGGACCACTTTGTACAAGAAAGCTGGGTATTAGATTCTCTTGGCACT |  |
| **11** | AT5g03350:OE-seq | Forward | ATGAAGATTCATAAACTC | pDONR207::AT5g03350:CDS construct sequencing |
|  |  | Reverse | TTAGATTCTCTTGGCACT |  |
| **12** | *hpt* | Forward | ATTGGGGAGTTTAGCGAGAG | Transformation confirmation and homozygosity test of overexpression line |
|  |  | Reverse | ATCGGCGAGTACTTCTACAC |  |
| **13** | *4hppd* (AT1G06570) | Forward | TCCGATTCCTTTTCACTGCT | homozygosity test of overexpression line |
|  |  | Reverse | TCTGGTAGTAAGTAGGCGGA |  |
| **14** | SALK_036814.56.00.x | SALK_036814.56.00.x Forward | TTGGGATGCAAAGCAAATTAC | Genotyping of AT5G03350 mutants |
|  |  | SALK_036814.56.00.x Reverse | CTTTCTCAGCAACAACGGAAG |  |
| **15** | LBP1.3 | LBP1.3 | ATTTTGCCGATTTCGGAAC | Genotyping of AT5G03350 mutants |

**Table S1** List of primers used in the current study
