# Supplementary material for: In silico characterization, molecular phylogeny, and expression profiling of genes encoding legume lectin-like proteins under various abiotic stresses in Arabidopsis thaliana
Source: BMC Genomics. 2022 Jun 29;23:480. doi: 10.1186/s12864-022-08708-0 (PMC9241310; doi:10.1186/s12864-022-08708-0)
Supplement: Supplementary file 3 — Additional file 3: Fig. S2. Represents the steps of overexpression line (At5g03350::overexpression) creation using gateway cloning technology followed by Agrobacterium mediated transformation. [file 12864_2022_8708_MOESM3_ESM.pptx]

## Slide 1
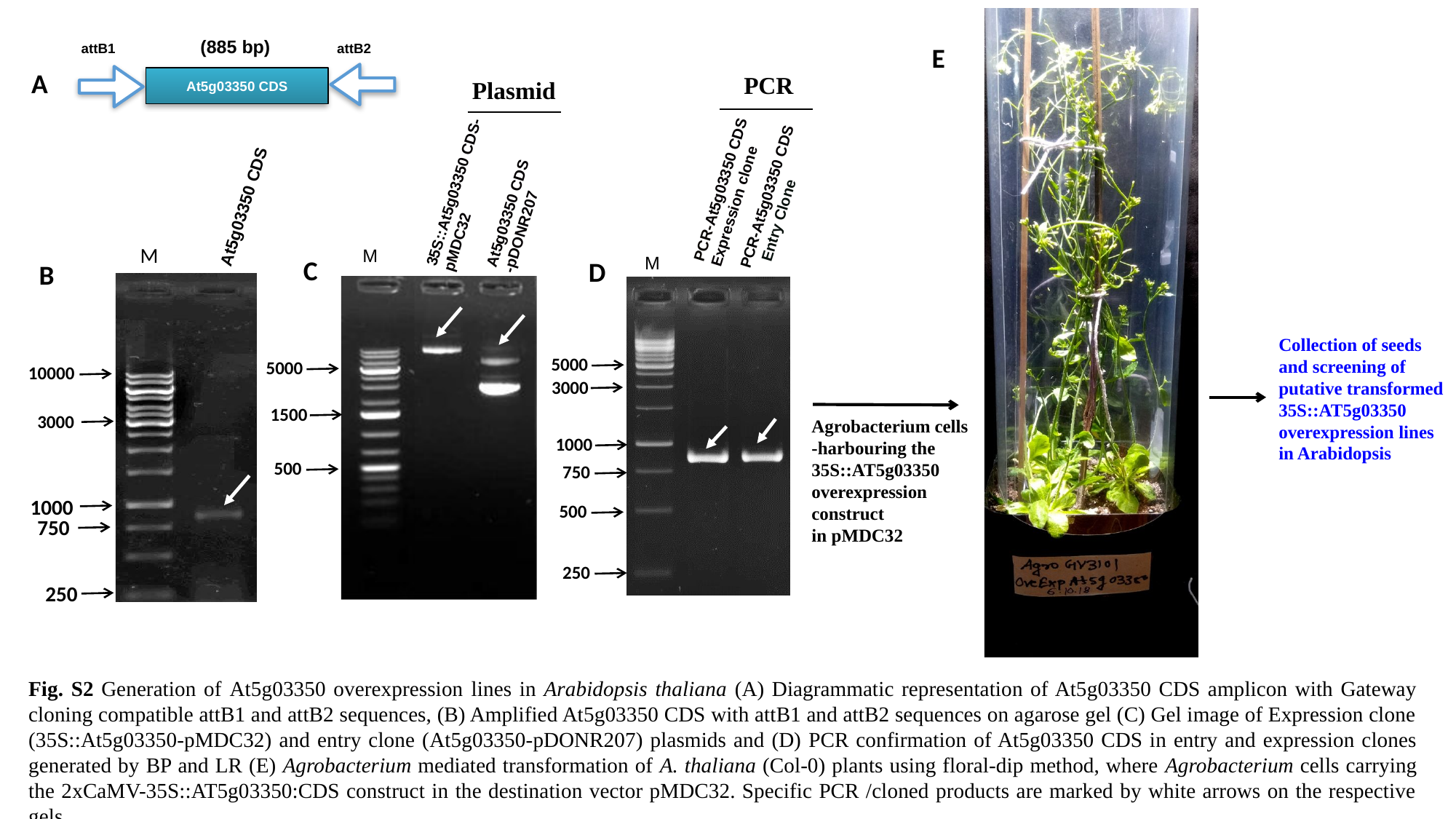

(885 bp)
attB1
attB2
At5g03350 CDS
E
A
PCR
Plasmid
PCR-At5g03350 CDS
Expression clone
At5g03350 CDS
-pDONR207
PCR-At5g03350 CDS
 Entry Clone
35S::At5g03350 CDS-pMDC32
At5g03350 CDS
M
M
M
C
D
B
Collection of seeds and screening of putative transformed 35S::AT5g03350 overexpression lines in Arabidopsis
5000
5000
10000
3000
1500
3000
Agrobacterium cells -harbouring the 35S::AT5g03350 overexpression construct
in pMDC32
1000
500
750
1000
500
750
250
250
Fig. S2 Generation of At5g03350 overexpression lines in Arabidopsis thaliana (A) Diagrammatic representation of At5g03350 CDS amplicon with Gateway cloning compatible attB1 and attB2 sequences, (B) Amplified At5g03350 CDS with attB1 and attB2 sequences on agarose gel (C) Gel image of Expression clone (35S::At5g03350-pMDC32) and entry clone (At5g03350-pDONR207) plasmids and (D) PCR confirmation of At5g03350 CDS in entry and expression clones generated by BP and LR (E) Agrobacterium mediated transformation of A. thaliana (Col-0) plants using floral-dip method, where Agrobacterium cells carrying the 2xCaMV-35S::AT5g03350:CDS construct in the destination vector pMDC32. Specific PCR /cloned products are marked by white arrows on the respective gels
