# Supplementary material for: In silico characterization, molecular phylogeny, and expression profiling of genes encoding legume lectin-like proteins under various abiotic stresses in Arabidopsis thaliana
Source: BMC Genomics. 2022 Jun 29;23:480. doi: 10.1186/s12864-022-08708-0 (PMC9241310; doi:10.1186/s12864-022-08708-0)
Supplement: Supplementary file 6 — Additional file 6: Fig. S3. Represents the steps for PCR-based genotyping for At5g03350:: overexpression line. [file 12864_2022_8708_MOESM6_ESM.pptx]

## Slide 1
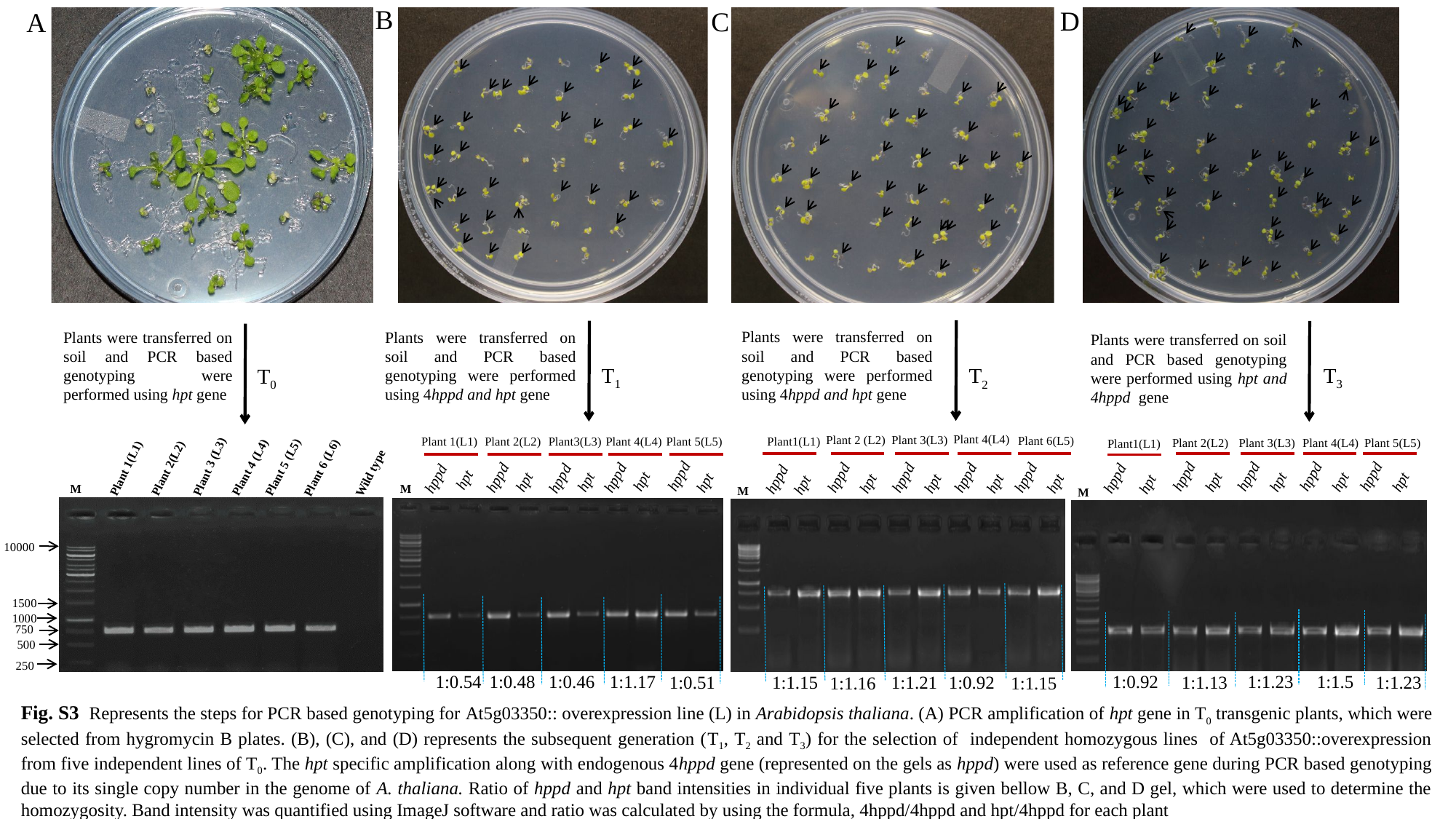

B
D
C
A
Plants were transferred on soil and PCR based genotyping were performed using 4hppd and hpt gene
Plants were transferred on soil and PCR based genotyping were performed using hpt gene
Plants were transferred on soil and PCR based genotyping were performed using 4hppd and hpt gene
Plants were transferred on soil and PCR based genotyping were performed using hpt and 4hppd gene
T1
T3
T2
T0
Plant 4(L4)
Plant 3(L3)
Plant 2 (L2)
Plant 6(L5)
Plant 1(L1)
Plant3(L3)
Plant 4(L4)
Plant 2(L2)
Plant 5(L5)
Plant1(L1)
Plant 4(L4)
Plant 3(L3)
Plant 5(L5)
Plant 2(L2)
Plant1(L1)
Plant 4 (L4)
Plant 6 (L6)
Plant 5 (L5)
Plant 1(L1)
Plant 3 (L3)
Plant 2(L2)
Wild type
hppd
hppd
hppd
hppd
hppd
hppd
hppd
hppd
hppd
hppd
hppd
hppd
hppd
hppd
hppd
hpt
hpt
hpt
hpt
hpt
hpt
hpt
hpt
hpt
hpt
hpt
hpt
hpt
hpt
hpt
M
M
M
M
10000
1500
1000
750
500
250
1:1.17
1:0.46
1:0.54
1:0.48
1:1.5
1:1.23
1:0.92
1:1.13
1:0.51
1:1.23
1:0.92
1:1.21
1:1.15
1:1.16
1:1.15
Fig. S3 Represents the steps for PCR based genotyping for At5g03350:: overexpression line (L) in Arabidopsis thaliana. (A) PCR amplification of hpt gene in T0 transgenic plants, which were selected from hygromycin B plates. (B), (C), and (D) represents the subsequent generation (T1, T2 and T3) for the selection of independent homozygous lines of At5g03350::overexpression from five independent lines of T0. The hpt specific amplification along with endogenous 4hppd gene (represented on the gels as hppd) were used as reference gene during PCR based genotyping due to its single copy number in the genome of A. thaliana. Ratio of hppd and hpt band intensities in individual five plants is given bellow B, C, and D gel, which were used to determine the homozygosity. Band intensity was quantified using ImageJ software and ratio was calculated by using the formula, 4hppd/4hppd and hpt/4hppd for each plant
