# Supplementary material for: In silico characterization, molecular phylogeny, and expression profiling of genes encoding legume lectin-like proteins under various abiotic stresses in Arabidopsis thaliana
Source: BMC Genomics. 2022 Jun 29;23:480. doi: 10.1186/s12864-022-08708-0 (PMC9241310; doi:10.1186/s12864-022-08708-0)
Supplement: Supplementary file 7 — Additional file 7: Fig. S4. Steps of PCR-based genotypic analysis of AT5g03350 gene-specific T-DNA insertion line. [file 12864_2022_8708_MOESM7_ESM.pptx]

## Slide 1
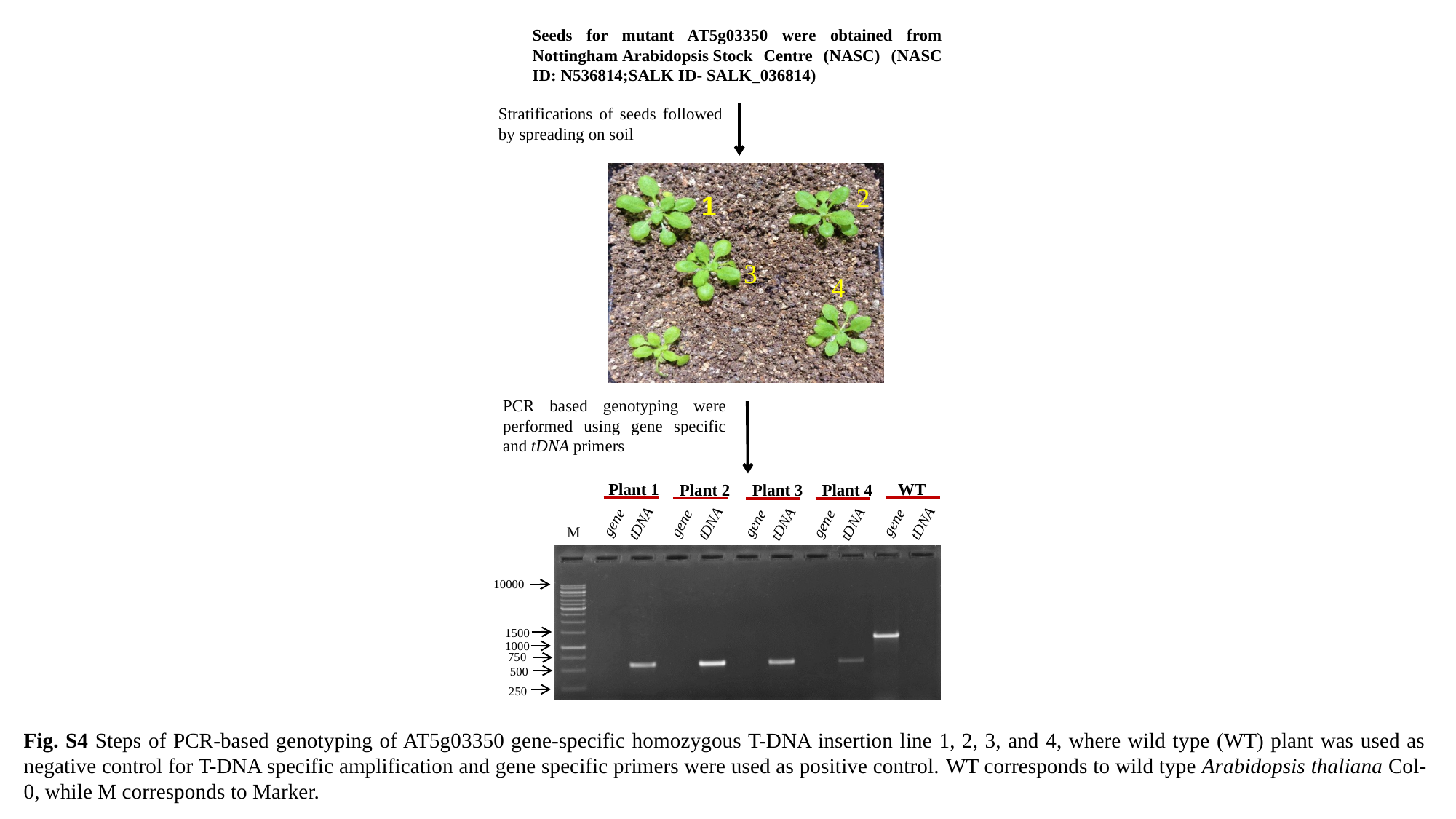

Seeds for mutant AT5g03350 were obtained from Nottingham Arabidopsis Stock Centre (NASC) (NASC ID: N536814;SALK ID- SALK_036814)
Stratifications of seeds followed by spreading on soil
2
1
3
4
PCR based genotyping were performed using gene specific and tDNA primers
Plant 1
gene
tDNA
WT
gene
tDNA
Plant 2
gene
tDNA
Plant 3
gene
tDNA
Plant 4
gene
tDNA
M
10000
1500
1000
750
500
250
Fig. S4 Steps of PCR-based genotyping of AT5g03350 gene-specific homozygous T-DNA insertion line 1, 2, 3, and 4, where wild type (WT) plant was used as negative control for T-DNA specific amplification and gene specific primers were used as positive control. WT corresponds to wild type Arabidopsis thaliana Col-0, while M corresponds to Marker.
