# Supplementary material for: In silico characterization, molecular phylogeny, and expression profiling of genes encoding legume lectin-like proteins under various abiotic stresses in Arabidopsis thaliana
Source: BMC Genomics. 2022 Jun 29;23:480. doi: 10.1186/s12864-022-08708-0 (PMC9241310; doi:10.1186/s12864-022-08708-0)
Supplement: Supplementary file 8 — Additional file 8: Fig. S5. Peptide sequence alignment of AtLLPs showing the presence of highly conserved lectin-binding domain and less conserved signal peptide. [file 12864_2022_8708_MOESM8_ESM.docx]

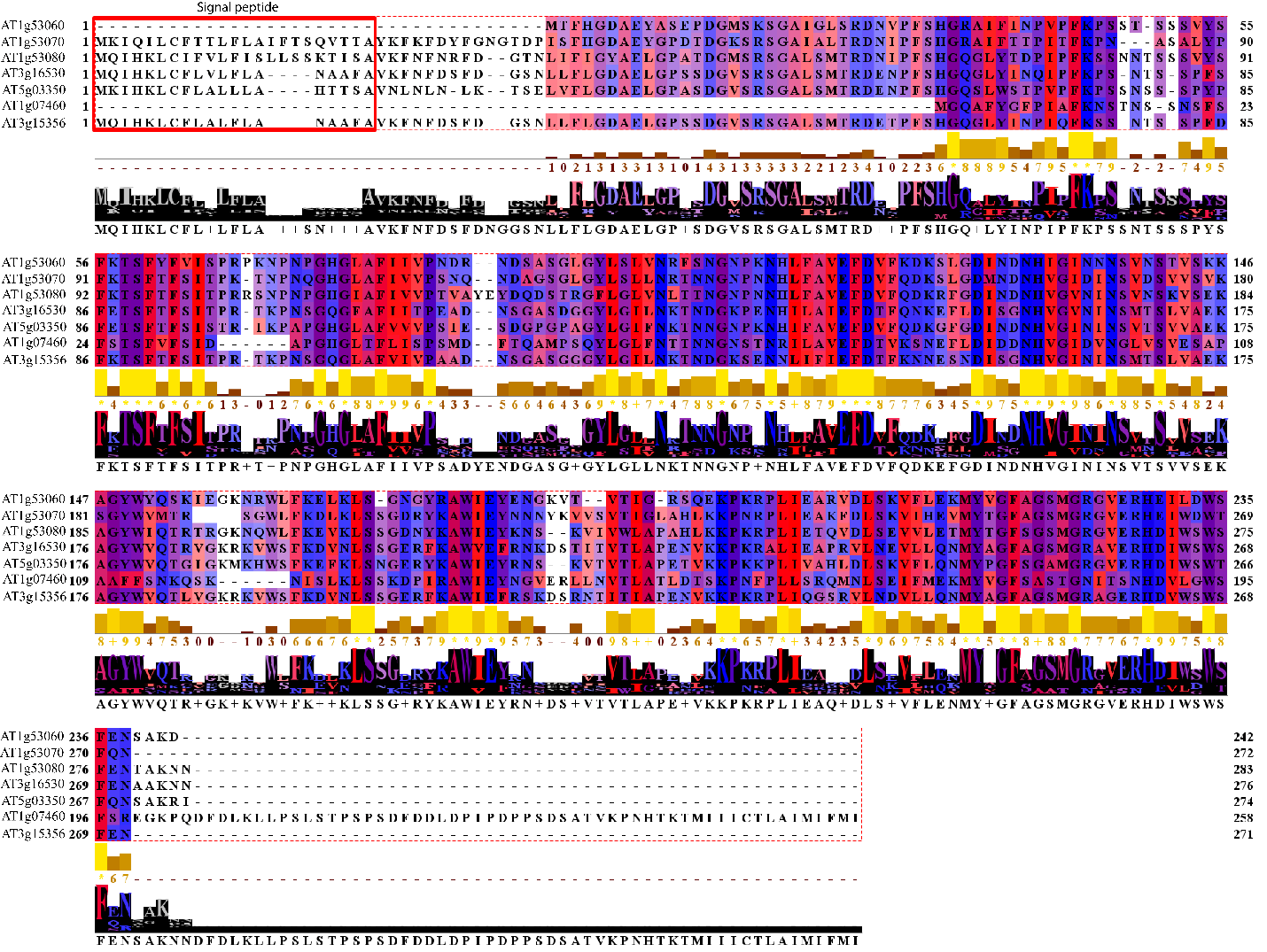


**Fig. S5** Peptide sequence alignment of AtLLPs showing the presence of highly conserved lectin-binding domain and less conserved signal peptide. The red-box represents the signal peptide. Conserved consensus sequences are represented as weblogo, height of the stack in weblogo and size of the bar are proportional to the conservation. Asterisk (*) marked below the conservation bars represent fully conserved amino acid residues in the sequences. The amino acid residues are colored on basis of their conservation with 10% cut-off.
