# Supplementary material for: In silico characterization, molecular phylogeny, and expression profiling of genes encoding legume lectin-like proteins under various abiotic stresses in Arabidopsis thaliana
Source: BMC Genomics. 2022 Jun 29;23:480. doi: 10.1186/s12864-022-08708-0 (PMC9241310; doi:10.1186/s12864-022-08708-0)
Supplement: Supplementary file 9 — Additional file 9: Table S2. Physicochemical properties of AtLLPs. [file 12864_2022_8708_MOESM9_ESM.docx]

**Table S2** Physicochemical properties of AtLLPs

| **S. N.** | **AGI** | **AA** | **MW**  **(KDa)** | **pI** | **Instability index** | **Aliphatic index** | **Grand average of hydropathicity (GRAVY)** |
| --- | --- | --- | --- | --- | --- | --- | --- |
| 1 | AT1g53060 | 242 | 27.01 | 9.34 | 34.43 | 69.26 | -0.520 |
| 2 | AT1g53070 | 272 | 30.38 | 8.49 | 14.50 | 75.26 | -0.259 |
| 3 | AT1g53080 | 283 | 31.83 | 9.28 | 28.26 | 75.72 | -0.330 |
| 4 | AT3g16530 | 276 | 30.51 | 6.98 | 32.51 | 73.88 | -0.289 |
| 5 | AT5g03350 | 274 | 30.16 | 9.39 | 30.04 | 77.19 | -0.244 |
| 6 | AT1g07460 | 258 | 28.36 | 4.80 | 36.14 | 80.50 | -0.088 |
| 7 | AT3g15356 | 271 | 29.75 | 8.91 | 32.61 | 73.43 | -0.290 |

**Footnote** AA corresponds to amino acid residues, MW to molecular weight, KDa to killo Dalton**,** and pI to isoelectric point
