# Supplementary material for: In silico characterization, molecular phylogeny, and expression profiling of genes encoding legume lectin-like proteins under various abiotic stresses in Arabidopsis thaliana
Source: BMC Genomics. 2022 Jun 29;23:480. doi: 10.1186/s12864-022-08708-0 (PMC9241310; doi:10.1186/s12864-022-08708-0)
Supplement: Supplementary file 10 — Additional file 10: Table S3. Predicted and experimentally confirmed subcellular location of AtLLPs. [file 12864_2022_8708_MOESM10_ESM.docx]

**Table S3** Predicted and experimentally confirmed subcellular location of *AtLLPs*

| **S. N.** | ***AtLLPs*** | **SUBA consensus** | **Predictions** | **Confirmed by MS/MS** |
| --- | --- | --- | --- | --- |
| 1 | AT1G53060 | Cytosol | Extracellular  Cytosol  Mitochondria  Nucleus  Plastid |  |
| 2 | AT1G53070 | Extracellular | Endoplasmic reticulum  Extracellular  Cytosol  Golgi  Plastid | Cytosol  Extracellular |
| 3 | AT1G53080 | Extracellular | Endoplasmic reticulum  Mitochondria  Extracellular  Plasma membrane  Cytosol  Golgi  Plastid | Extracellular |
| 4 | AT3G16530 | Extracellular | Endoplasmic reticulum  Extracellular  Plasma membrane  Nucleus  Cytosol  Golgi | Extracellular  Nucleus  Plasma membrane |
| 5 | AT5G03350 | Extracellular | Endoplasmic reticulum  Plasma membrane  Cytosol  Nucleus  Golgi  Extracellular | Extracellular  Plastid |
| 6 | AT1g07460 | Plastid | Endoplasmic reticulum  Extracellular  Mitochondria  Plastid  Cytosol  Golgi  Plasma membrane  Nucleus |  |
| 7 | AT3g15356 | Extracellular | Cytosol  Nucleus  Extracellular  Endoplasmic reticulum  Golgi | Extracellular  Mitochondria  Plasma membrane  Plastid |
