# Supplementary material for: In silico characterization, molecular phylogeny, and expression profiling of genes encoding legume lectin-like proteins under various abiotic stresses in Arabidopsis thaliana
Source: BMC Genomics. 2022 Jun 29;23:480. doi: 10.1186/s12864-022-08708-0 (PMC9241310; doi:10.1186/s12864-022-08708-0)
Supplement: Supplementary file 11 — Additional file 11: Table S4. Estimation of divergence time of Arabidopsis thaliana LLP gene family and its orthologs in Arabidopsis lyrata, Brassica rapa, Solanum lycopersicum, Zea mays, Selaginella moellendorffii, Physcomitrium patens, and Chlamydomonas reinhardtii. [file 12864_2022_8708_MOESM11_ESM.doc]

**Table S4** Estimation of divergence time of *Arabidopsis thaliana* *LLP* gene family and its orthologs in *Arabidopsis lyrata*, *Brassica rapa*, *Solanum lycopersicum*, *Zea mays*, *Selaginella moellendorffii*, *Physcomitrium patens*, and *Chlamydomonas reinhardtii*.

| **S. N.** | **Ortholog Pairs** | **S** | **N** | **dS** | **dN** | **dN/dS** | **Divergence Time** |
| --- | --- | --- | --- | --- | --- | --- | --- |
|  | *A. thaliana- C. reinhardtii* |  |  |  |  |  |  |
| 1 | AT1G53060.1-CHLRE_11g475000v5 | 100.9 | 415.1 | 61.7567 | 1.1496 | 0.0186 | 190.0206154 |
| 2 | AT1G53070.1- CHLRE_11g475000v5 | 139.4 | 625.6 | 65.8005 | 1.2383 | 0.0188 | 202.4630769 |
| 3 | AT1G53080.1- CHLRE_11g475000v5 | 150.7 | 665.3 | 65.0613 | 1.2028 | 0.0185 | 200.1886154 |
| 4 | AT3G16530.1- CHLRE_11g475000v5 | 133.8 | 607.2 | 67.6161 | 0.9640 | 0.0143 | 208.0495385 |
| 5 | AT5G03350.1- CHLRE_11g475000v5 | 143.9 | 627.1 | 64.4614 | 1.1900 | 0.0185 | 198.3427692 |
| 6 | AT3G15356.1- CHLRE_11g475000v5 | 133.7 | 607.3 | 67.1566 | 1.0747 | 0.0160 | 206.6378462 |
|  | *A. thaliana- P. patens* |  |  |  |  |  |  |
| 7 | AT1G53060.1-At1g53060.1 [*P*. *patens*] | 164.6 | 489.4 | 32.4334 | 0.6736 | 0.0208 | 99.79507692 |
| 8 | AT1G53070.1-lectin-like protein At1g53070 [*P*. *patens*] | 165.3 | 587.7 | 56.3636 | 0.8043 | 0.0143 | 173.4264615 |
| 9 | AT1G53080.1- L-type lectin-domain containing receptor kinase S.7 [*P*. *patens*] | 172.5 | 580.5 | 54.0152 | 0.8104 | 0.0150 | 166.2006154 |
| 10 | AT3G16530.1-L-type lectin-domain containing receptor kinase S.7 [*P*. *patens*] | 167.7 | 585.3 | 16.6362 | 0.7696 | 0.0463 | 51.18830769 |
| 11 | AT5G03350.1- L-type lectin-domain containing receptor kinase S.7 [*P*. *patens*] | 171.4 | 572.6 | 52.6386 | 0.8849 | 0.0168 | 161.9649231 |
| 12 | AT3G15356.1- L-type lectin-domain containing receptor kinase S.7 [*P*. *patens*] | 163.8 | 574.2 | 18.1232 | 0.8382 | 0.0463 | 55.76369231 |
|  | *A. thaliana- S. moellendorffii* |  |  |  |  |  |  |
| 13 | AT1G53060.1- 30228 (*S*. *moellendorffii*) | 143.0 | 448.0 | 51.3237 | 0.7665 | 0.0149 | 157.9190769 |
| 14 | AT1G53070.1-135805 (*S*. *moellendorffii*) | 147.3 | 551.7 | 59.1815 | 0.6748 | 0.0114 | 182.0969231 |
| 15 | AT1G53080.1-135805 (*S*. *moellendorffii*) | 185.8 | 591.2 | 51.7857 | 0.8106 | 0.0157 | 159.3406154 |
| 16 | AT3G16530.1-172624 (*S*. *moellendorffii*) | 130.0 | 476.0 | 57.6935 | 0.7985 | 0.0138 | 177.5184615 |
| 17 | AT5G03350.1- 15696 (*S*. *moellendorffii*) | 139.0 | 479.0 | 20.5885 | 0.7170 | 0.0348 | 63.34923077 |
| 18 | AT3G15356.1- 30228 (*S*. *moellendorffii*) | 133.0 | 446.0 | 53.8615 | 0.8174 | 0.0152 | 165.7276923 |
|  | *A. thaliana- Z. mays* |  |  |  |  |  |  |
| 19 | AT1G53060.1- Zm00001d043781 | 151.1 | 547.9 | 57.2181 | 0.8047 | 0.0141 | 17.60556923 |
| 20 | AT1G53070.1- Zm00001d053322 | 160.0 | 623.0 | 60.5660 | 0.7795 | 0.0129 | 18.63569231 |
| 21 | AT1G53080.1- Zm00001d028455 | 176.2 | 618.8 | 55.8754 | 0.7902 | 0.0141 | 17.19243077 |
| 22 | AT3G16530.1- Zm00001d028454 | 161.9 | 618.1 | 59.8067 | 0.7436 | 0.0124 | 18.40206154 |
| 23 | AT5G03350.1- Zm00001d024637 | 189.1 | 605.9 | 52.0132 | 0.8269 | 0.0159 | 16.00406154 |
| 24 | AT3G15356.1- Zm00001d043781 | 172.2 | 592.8 | 55.2003 | 0.7369 | 0.0133 | 16.98470769 |
|  | *A. thaliana-* *S. lycopersicum* |  |  |  |  |  |  |
| 25 | AT1G53060.1- Solyc02g078170.3.1 | 154.0 | 566.0 | 58.4388 | 0.6370 | 0.0109 | 17.98116923 |
| 26 | AT1G53070.1-Solyc07g026800.1.1 | 171.7 | 602.3 | 56.0766 | 0.7202 | 0.0128 | 17.25433846 |
| 27 | AT1G53080.1-Solyc03g112310.1.1 | 191.8 | 615.2 | 52.1620 | 0.7871 | 0.0151 | 16.04984615 |
| 28 | AT3G16530.1-Solyc03g080060.1.1 | 166.5 | 571.5 | 54.3500 | 0.9576 | 0.0176 | 16.72307692 |
| 29 | AT5G03350.1-Solyc09g011840.1.1 | 145.6 | 577.4 | 60.6527 | 0.9828 | 0.0162 | 18.66236923 |
| 30 | AT3G15356.1- Solyc02g078170.1.1 | 171.8 | 608.2 | 12.9771 | 0.7180 | 0.0553 | 3.992953846 |
|  | *A. thaliana- B. rapa* |  |  |  |  |  |  |
| 31 | AT1G53060.1- lectin-like protein At1g53070 [*B*. *rapa*] | 170.8 | 528.2 | 1.0488 | 0.2546 | 0.2427 | 3.227076923 |
| 32 | AT1G53070.1- lectin-like protein At1g53070 [*B*. *rapa*] | 188.1 | 627.9 | 0.5627 | 0.1228 | 0.2182 | 1.731384615 |
| 33 | AT1G53080.1- lectin-like protein At1g53080 [*B*. *rapa*] | 193.2 | 649.8 | 0.4229 | 0.0604 | 0.1428 | 1.301230769 |
| 34 | AT3G16530.1- lectin-like protein At3g16530 [*B*. *rapa*] | 199.4 | 628.6 | 0.6038 | 0.0947 | 0.1568 | 1.857846154 |
| 35 | AT5G03350.1- lectin-like protein [*B*. *rapa*] | 215.0 | 592.0 | 0.5730 | 0.1357 | 0.2368 | 1.763076923 |
| 36 | AT3G15356.1- lectin-like protein [*B*. *rapa*] | 198.7 | 599.3 | 0.8642 | 0.2812 | 0.3254 | 2.659076923 |
|  | *A. thaliana- A. lyrata* |  |  |  |  |  |  |
| 37 | AT1G53060.1- At1g53060 (LOC9330485) | 189.4 | 536.6 | 0.1467 | 0.0507 | 0.3459 | 0.451384615 |
| 38 | AT1G53070.1- At1g53070 (LOC9327788) | 219.2 | 596.8 | 0.1076 | 0.0188 | 0.1749 | 0.331076923 |
| 39 | AT1G53080.1- At1g53080 (LOC9327789) | 205.1 | 637.9 | 0.1723 | 0.0405 | 0.2348 | 0.530153846 |
| 40 | AT3G16530.1- At3g16530 (LOC9321213) | 220.0 | 608.0 | 0.0555 | 0.0149 | 0.2690 | 0.170769231 |
| 41 | AT5G03350.1- lectin-like protein LEC (LOC9321147) | 195.9 | 608.1 | 0.5638 | 0.2613 | 0.4634 | 1.734769231 |
| 42 | AT3G15356.1- lectin-like protein LEC (LOC9321147) | 199.7 | 613.3 | 0.1369 | 0.0298 | 0.2176 | 0.421230769 |

S corresponds to number of synonymous sites, N corresponds to number of non-synonymous sites, dS correspond to synonymous substitution rate (synonymous substitutions per synonymous site), dN corresponds to non-synonymous substitution rate (non-synonymous substitutions per non-synonymous site), and dN/dS corresponds to dN/dS ratio
