# Supplementary material for: In silico characterization, molecular phylogeny, and expression profiling of genes encoding legume lectin-like proteins under various abiotic stresses in Arabidopsis thaliana
Source: BMC Genomics. 2022 Jun 29;23:480. doi: 10.1186/s12864-022-08708-0 (PMC9241310; doi:10.1186/s12864-022-08708-0)
Supplement: Supplementary file 14 — Additional file 14: Table S7. Number of predicted transcription factors (TFs), which can bind cognate cis-regulatory elements (CREs) in the promoter region of AtLLPs, as well as the list of TFs not included in this study and the number of TFs that are significantly correlated under different stress conditions in both root and shoot tissues. [file 12864_2022_8708_MOESM14_ESM.docx]

**Table S7** Number of predicted transcription factors (TFs), which can bind cognate *cis*-regulatory elements (CREs) in the promoter region of *AtLLPs*, as well as the list of TFs not included in this study and the number of TFs that are significantly correlated under different stress conditions in both root and shoot tissues.

| **S. N.** | ***AtLLPs*** | **Total number of TFs identified in promoter analysis** | **Expression data not available** | **Number of TFs which showed significant correlation with tested *AtLLPs*** | |
| --- | --- | --- | --- | --- | --- |
|  |  |  |  | **Root** | **Shoot** |
| 1 | AT1g53060 | 63 | AT3g48430 | 16 | 26 |
| 2 | AT1g53070 | 51 | AT1g09540, AT1g19790, AT4g38910 | 31 | 17 |
| 3 | AT1g53080 | 38 |  | 13 | 8 |
| 4 | AT3g16530 | 120 | AT1g03800, AT1g45249, AT1g67260, AT2g21900, AT3g45150, AT3g46090, AT4g21040, AT5g01900 | 53 | 44 |
| 5 | AT5g03350 | 84 | AT1g09540, AT1g18960, AT2g46770, AT3g46090, AT4g21330, AT4g38910, AT5g56840, AT5g61270 | 39 | 27 |
| 6 | AT1g07460 | 93 | At1g02230  At1g31140  At1g32510  At2g46770  At3g46090  At3g51060  At4g23550  At5g41570 | 26 | 22 |
| 7 | AT3g15356 | 161 | NA | NA | NA |
